# The flavour of grape colour: anthocyanin content tunes aroma precursor composition by altering the berry microenvironment

**DOI:** 10.1093/jxb/erad223

**Published:** 2023-06-09

**Authors:** Maite Rodríguez-Lorenzo, Nuria Mauri, Carolina Royo, José L Rambla, Gianfranco Diretto, Olivia Demurtas, Ghislaine Hilbert, Christel Renaud, Vanessa Tobar, Joaquín Huete, Serge Delrot, Antonio Granell, José Miguel Martínez-Zapater, Pablo Carbonell-Bejerano

**Affiliations:** Instituto de Ciencias de la Vid y del Vino, ICVV, CSIC - Universidad de La Rioja - Gobierno de La Rioja, 26007 Logroño, Spain; Instituto de Ciencias de la Vid y del Vino, ICVV, CSIC - Universidad de La Rioja - Gobierno de La Rioja, 26007 Logroño, Spain; Instituto de Ciencias de la Vid y del Vino, ICVV, CSIC - Universidad de La Rioja - Gobierno de La Rioja, 26007 Logroño, Spain; Instituto de Biología Molecular y Celular de Plantas, IBMCP, CSIC - Universidad Politécnica de Valencia, 46011 Valencia, Spain; Universitat Jaume I, Departamento de Biología, Bioquímica y Ciencias Naturales, 12071 Castellón de la Plana, Spain; Italian National Agency for New Technologies Energy and Sustainable Development, Casaccia Research Centre, 00123 Rome, Italy; Italian National Agency for New Technologies Energy and Sustainable Development, Casaccia Research Centre, 00123 Rome, Italy; EGFV, Bordeaux Sciences Agro, INRA - Université de Bordeaux, ISVV, 33140 Villenave d’Ornon, France; EGFV, Bordeaux Sciences Agro, INRA - Université de Bordeaux, ISVV, 33140 Villenave d’Ornon, France; Servicio de Información Agroclimática de La Rioja (SIAR). Consejería de Agricultura, Ganadería y Medio Ambiente, Gobierno de La Rioja, 26007 Logroño, Spain; Servicio de Información Agroclimática de La Rioja (SIAR). Consejería de Agricultura, Ganadería y Medio Ambiente, Gobierno de La Rioja, 26007 Logroño, Spain; EGFV, Bordeaux Sciences Agro, INRA - Université de Bordeaux, ISVV, 33140 Villenave d’Ornon, France; Instituto de Biología Molecular y Celular de Plantas, IBMCP, CSIC - Universidad Politécnica de Valencia, 46011 Valencia, Spain; Instituto de Ciencias de la Vid y del Vino, ICVV, CSIC - Universidad de La Rioja - Gobierno de La Rioja, 26007 Logroño, Spain; Instituto de Ciencias de la Vid y del Vino, ICVV, CSIC - Universidad de La Rioja - Gobierno de La Rioja, 26007 Logroño, Spain; University of Trento, Italy

**Keywords:** Anthocyanin, aroma precursor, berry microenvironment, flavonol trihydroxylation, grape colour, metabolomics, MYBA transcription factors, somatic variation, transcriptomics, *Vitis vinifera*, volatiles

## Abstract

Anthocyaninless (white) instead of black/red (coloured) fruits develop in grapevine cultivars without functional *VviMYBA1* and *VviMYBA2* genes, and this conditions the colour of wines that can be produced. To evaluate whether this genetic variation has additional consequences on fruit ripening and composition, we performed comparisons of microenvironment, transcriptomics, and metabolomics of developing grapes between near-isogenic white- and black-berried somatic variants of Garnacha and Tempranillo cultivars. Berry temperature was as much as 3.5 ºC lower in white- compared to black-berried Tempranillo. An RNA-seq study combined with targeted and untargeted metabolomics revealed that ripening fruits of white-berried variants were characterized by the up-regulation of photosynthesis-related and other light-responsive genes and by their higher accumulation of specific terpene aroma precursors, fatty acid-derived aldehyde volatiles, and phenylpropanoid precursor amino acids. MYBA1-MYBA2 function proved essential for flavonol trihydroxylation in black-berried somatic variants, which were also characterized by enhanced expression of pathogen defence genes in the berry skin and increased accumulation of C_6_-derived alcohol and ester volatiles and γ-aminobutyric acid. Collectively, our results indicate that anthocyanin depletion has side-effects on grape composition by altering the internal microenvironment of the berry and the partitioning of the phenylpropanoid pathway. Our findings show how fruit colour can condition other fruit features, such as flavour potential and stress homeostasis.

## Teaser

Specific flavour-related biochemical composition and signalling features of white grapes are associated with variations in the berry microenvironment due to the absence of anthocyanin sunscreen pigments in the berry skin.

## Introduction

Dark colour in mature berries of cultivated grapevines (*Vitis vinifera* ssp. *vinifera*) results from the biosynthesis and vacuolar accumulation of anthocyanins, most commonly in berry skin cells (Boss *et al.*, 1996). Berry anthocyanins are considered to play important biological roles in the attraction of vertebrates for seed dispersal (Gautier-Hion *et al.*, 1985), as well as in the protection of inner tissues and embryos from photo-oxidative stress (Winkel, 2004). Wild species of the genus *Vitis* and *V. vinifera* ssp. *sylvestris*, the putative wild ancestor of cultivated grapevines, generally produce small, dark black/purple berries, suggesting that anthocyanin pigmentation is an adaptive trait in wild *Vitis* populations (Olmo, 1976; Cadle-Davidson and Owens, 2008). Berry colour diversification due to decreased anthocyanin accumulation was probably a derived trait selected during grapevine domestication (Zohary and Hopf, 2000), and is related to grape cultivars displaying a wide range of berry colour from black/purple to anthocyaninless ‘white’ (Fournier-Level *et al.*, 2010). Berry colour is an important trait that traditionally contributes to wine diversification. Chemical residues in ancient amphorae suggest that Egyptians were already growing white-berried grapevines for white wine making >3000 years ago (Guasch-Jané *et al.*, 2006).

Variation for grape colour in grapevine cultivars is genetically determined by a major quantitative trait locus (QTL) located on linkage group 2 (LG2) (Fischer *et al.*, 2004; Doligez *et al.*, 2006a; Fournier-Level *et al.*, 2009, 2010). This locus overlaps with a cluster of tandemly duplicated *MYBA* genes (Wong *et al.*, 2016), including *VviMYBA1* and *VviMYBA2* that are essential for anthocyanin accumulation in grape berries. MYBA1 and MYBA2 trigger the expression of target genes such as *VviUFGT*, which in turn encodes the UDP-glucose:flavonoid 3-O-glucosyltransferase enzyme that limits anthocyanin production (Kobayashi *et al.*, 2002; Walker *et al.*, 2007). Black/red-berried cultivars carry at least one functional allele of the grape-colour locus (with functional *VviMYBA1* and *VviMYBA2*), whereas most analysed white-berried cultivars are homozygous for the canonical null allele that carries recessive mutations knocking out both genes (Kobayashi *et al.*, 2004; Walker *et al.*, 2007). In addition, black-berried cultivars heterozygous for that null allele occasionally display decreased grape-colour somatic variants with either red/grey berries when the deletion of the functional allele occupies only the L2 meristem cell layer or white grapes if it extends to both L1+L2 (Carbonell-Bejerano *et al.*, 2019).

Grape anthocyanins play a crucial role in wine organoleptic attributes. In addition to their colour contribution, they display complex interactions with other compounds that determine the in-mouth feel of wines (Vidal *et al.*, 2004). However, the relationship between the absence of anthocyanins and other grape composition features has only rarely been studied. While no systematic report has focused on the effects of grape colour on aroma-related compounds, at the phenolic composition level a few studies have shown that grapes of white-berried genotypes tend to display certain features, including higher accumulation of phenylalanine, hydroxycinnamic acids, and flavonols (Degu *et al.*, 2015; Ferreira *et al.*, 2017), as well as an increased proportion of dihydroxylated to trihydroxylated flavonoid forms (Mattivi *et al.*, 2006; Ferreira *et al.*, 2016). This variation correlates with differential regulation of phenylpropanoid pathway genes between grapes of white and anthocyanin-pigmented genotypes and enhanced transcriptome responses to radiation in white grapes (Rinaldo *et al.*, 2015; Massonnet *et al.*, 2017; Ferreira *et al.*, 2019; Zhang *et al*., 2021, Preprint). While white grapes and wines tend to display more flowery and white-fruit notes (Petronilho *et al.*, 2020), additional systematic analyses are required to determine whether their characteristic flavour properties are related to the absence of anthocyanins or are just due to independent varietal features selected for the production of different types of wine. Additional specific features of white and red wines arise from differences in the dedicated winemaking processes that avoid or favour the maceration of the must with the berry skins, respectively.

To understand whether and how fruit ripening and flavour-related composition are conditioned by genetic variation for grape colour, in this study we performed comparisons of berry microenvironment, transcriptomics, and metabolomics between near-isogenic white- and black-berried somatic variants. Our results uncover previously unknown effects of grape colour on the berry internal temperature and the accumulation of important grape and wine aroma precursor and signalling metabolites.

## Materials and methods

### Plant material

We used somatic mutant variants that share the same zygotic origin but differ in grape berry skin colour as a genetic tool to study side effects associated with skin colour variation in near-isogenic backgrounds. Specifically, we compared *Vitis vinifera* ssp. *vinifera* Tempranillo Blanco (TB) and Garnacha Blanca (GB) white-skinned berry somatic variants, which harbour deletions in the L1 and L2 meristem cell layers that fully eliminate functional alleles of *VviMYBA1* and *VviMYBA2* and hence the capacity for fruit anthocyanin pigmentation (Migliaro *et al.*, 2014; Carbonell-Bejerano *et al.*, 2017), to their black-skinned berry clonal ancestors Tempranillo Tinto and Garnacha Tinta, respectively. Tempranillo Tinto clone RJ51 (TT), Tempranillo Blanco unique clone (TB), Garnacha Tinta ICVV1608 accession (GT), and Garnacha Blanca ICVV1662 accession (GB) were grown in two adjacent plots belonging to the Grapevine Germplasm Collection of the Instituto de Ciencias de la Vid y del Vino (ICVV; ESP-217) at Finca La Grajera (Logroño, La Rioja, Spain). The selected GB and GT accessions originated from vineyards of the same geographic location (Bargota, Navarra, Spain). According to whole-genome sequencing comparisons, because of a simple deletion in the grape-colour locus, the GB ICVV-1662 accession is hemizygous only for another 33 genes in addition to *VviMYBA1* and *VviMYBA2* as compared to the GT ICVV-1608 accession (0.11% of genes annotated in the genome), with no other predicted loss-of-function allele remaining hemizygous apart from *VviMYBA2* (Royo *et al.*, 2019). Related to more complex genome rearrangements including translocations between LG2 and LG5, TB is hemizygous for another 311 genes compared to TT (1.0% of genes annotated in the genome), including 21 hemizygous genes with amino acid substitutions but with no other hemizygous gene with major effect variants predicted, in agreement with low heterozygosity in the TT cultivar for the affected region in LG2 (Carbonell-Bejerano *et al.*, 2017).

The experimental plots consisted of three interspersed TT and TB east–west oriented rows (>50 vines per row planted in 2002, located at 42°26´20.3938´´ N, 2°30’52.9165´´W), and single adjacent GT and GB north–south oriented rows (five plants per accession planted in 2008, located at 42°26´’16.4602´´N, 2°30´45.4428´´W). All plants were grafted on Richter-110 rootstock, trellised in a double cordon Royat system, and cultivated under the same agronomical practices. Removal of leaves covering grape bunches was performed from the onset of ripening. Tempranillo and Garnacha true-to-typeness of the four accessions was confirmed in gDNA extracted from young leaves using DNeasy Plant Mini Kit (Qiagen) by the analysis of nine microsatellite loci according to Ibanez *et al.* (2009).

### Berry temperature measurements

To measure berry temperature, Type T thermocouples prepared from copper–constantan cables (RS Components) were inserted into TB and TT grapes in adjacent vineyard rows, corresponding to one of the replicates used for the berry transcriptome and metabolite analyses. The Type T thermocouple tips were hand-prepared by welding the ends of the two cables, and the average length of the temperature sensory ends was 6 ± 1 mm. For each berry, the thermocouple welded end was inserted with the base crossing the skin and the tip reaching approximately to the centre of the flesh, so that estimated temperature according to the differential electrical current between the two thermocouple cables corresponded to temperature across the pericarp. The thermocouples were connected to an AM 16/32 Multiplexer and data were recorded by means of a CR10X Datalogger (both Campbell Scientific). Berry temperature was simultaneously recorded for TB and TT during 10 consecutive sunny days beginning on 16 August 2016, in nine already-ripening grapes (five from the north-facing and four from the south-facing sides of the row from different bunches in five different plants). In addition, berry temperature was registered for 4 consecutive post-veraison days (11–15 August) and 29 consecutive pre-harvest days (17 August–15 September) that included some cloudy and rainy days. During these periods, the thermocouples were moved to new berries in the same cluster when some berry shrinking or drying was observed. To simultaneously register air temperature, a T107 sensor (Campbell Scientific) was installed in the TT vineyard row at the canopy level and connected to the datalogger. Temperatures were recorded every 30 s and mean values per hour per sensor were determined. Mean berry temperature per side of row per hour was calculated for each accession.

### Fruit sampling

Berries at different developmental stages were collected for profiling of the transcriptome and metabolite composition (Supplementary Table S1). Independent plants (from different vineyard rows in the case of Tempranillo accessions) were considered as biological replicates, and three replicates per accession were collected for each stage. Each replicate consisted of berries from two or three different clusters of the same plant that were collected from sun-facing south or west sides of the vineyard rows for Tempranillo and Garnacha, respectively. Berries were sorted by density using a NaCl solution series to standardize the ripening status within and across accessions, at least in terms of sugar content (Carbonell-Bejerano *et al.*, 2016). Pre-veraison green fruits were collected based on hand touch and visual inspection of colour (hard and green berries). Thereafter, at each sampling stage from veraison (the onset of ripening), berries of the same density range were selected in all four accessions to standardize the ripening status for the veraison, pre-maturity, and maturity stages according to the density ranges described in Supplementary Table S1. The berries selected in the NaCl series were rinsed in water, dried with paper tissue, immediately frozen in liquid nitrogen, and stored in a freezer at –80 ºC until further transcriptome and metabolite analysis. A fraction of the fresh density-selected berries was directly used for quantification of total soluble solids (TSS) content using a WM-7 digital refractometer (ATAGO). For the transcriptomic analysis, pre-veraison (PV, hard green berries), veraison (V, soft berries collected when ~50% of berries were softened in the cluster), and pre-maturity (PM, mid-ripening) fruit samples were collected on warm sunny days during the 2016 season (Supplementary Table S1). To avoid circadian and environmental bias on each sampling date, each replicate was collected and fully processed one after another, and simultaneously for all four accessions. Each sample was processed from collection to freezing within 1 h, while the whole set of replicates was collected and frozen within 2 h on each sampling date (from 14.00 to 16.00 h). For metabolite composition, the same sampling stages that were analysed for transcriptomics in the 2016 season were collected in the 2017 season, plus additional berries at the maturity (M) stage collected at vineyard harvest in both the 2016 and 2017 seasons. The PM berries from 2016 that were used for transcriptomics were also used for phytohormone analysis (Supplementary Table S1). The PV, V, and PM pre-harvest samples from the 2016 season were subjected to RNA-seq to examine grape colour variation effects on berry development and ripening, whilst berry composition was quantified at the maturity stage in 2016 to examine possible consequences on organoleptic properties at the final harvest time. In addition, a large part of the metabolite families (sugars, organic acids, amino acids, flavonols, and anthocyanins) were quantified again in the 2017 season in the four developmental stages during which either RNA-seq or metabolite quantification had been conducted in the previous 2016 season (PV, V, PM, and M) to assess for intra- and inter-season consistency of the effects (Supplementary Table S1).

### Transcriptome RNA-seq analysis

#### RNA extraction and sequencing

The frozen sampled berries (10–15 per sample) were briefly thawed, then peeled and de-seeded. The berry flesh and skins were then separately homogenized in liquid nitrogen and submitted to total RNA extraction as described by Carbonell-Bejerano *et al.*, (2014b). Library preparation and high-throughput sequencing of RNA samples was performed at the Centre for Genomic Regulation (CRG, Barcelona, Spain) using 600 ng of total RNA. Illumina TruSeq Stranded mRNA technology was used to prepare a total of 72 libraries that were sequenced on a HiSeq2500 sequencer using V4 chemistry. An average of 32.71 ± 2.97 million of 2 × 125 bp stranded reads were produced per library.

#### Transcriptome-wide analysis of differential gene expression

After marking remaining adapter sequences using ‘MarkIlluminaAdapters’ in Picard-tools v.2.9.4. (http://broadinstitute.github.io/picard/), reads were mapped to the PN40024 12X.0 reference genome assembly (The French–Italian Public Consortium for Grapevine Genome Characterization, 2007) using a two-pass mode in HISAT2 v.2.1.0 (Kim *et al.*, 2015). Potential PCR duplicates were removed using ‘MarkDuplicates’ in Picard tools v.2.9.4 and only alignments that were pair concordant and unique (according to MAPQ≥30) were considered. Read-pairs mapping to exon regions in the grapevine 12X V1 gene annotations (Grimplet *et al.*, 2012) were counted using the ‘htseq-count tool’ of HTSeq v.0.11.1 (Anders *et al.*, 2015). Counts per gene were normalized and converted to fragments per kb per million counts (FPKM) using the ‘calcNormFactors’ and ‘rpkm’ functions in edgeR v.3.24.3 (Robinson *et al.*, 2010). A multidimensional scaling (MDS) plot from the normalized FPKM counts was produced using the ‘plotMDS’ function in edgeR.

In order to follow the progression of changes in gene expression during berry development and ripening, differential expression was analysed by comparisons of the full development and ripening time-course series including the PV, V, and PM stages between the white- and black-berried somatic variants. The white- versus black-berried somatic variants were independently compared for the time-course series of each cultivar and for each tissue, namely Tempranillo berry skin (TS), Tempranillo berry flesh (TF), Garnacha berry skin (GS), and Garnacha berry flesh (GF). In addition, the fold-change in expression was computed between the white- and the black-berried variants at each stage (PV, V, and PM) for each of the four developmental series (TS, TF, GS, and GF). Genes with either low expression levels or low magnitude of changes between the somatic variants were discarded: using the ‘topTags’ function in edgeR, for each of the four series only genes with mean FPKM ≥1 in at least one of the six samples of the same series (three stages × two somatic variants) and |fold-change|≥1.75 for white- versus black-berried pairwise comparison in at least one of the three stages of the series were further considered. In addition to these filters, statistically significant differences in gene expression between white- versus black-berried somatic variants along the developmental time-course series of the PV, V, and PM stages were analysed using maSigPro v.1.60.0 (Nueda *et al.*, 2014). For each of the TS, TF, GS, and GF series, genes fitting the model with *R*^2^≥0.6 and a false discovery rate (FDR) ≤0.05 in the maSigPro two-class time-course series (white- versus black-berried classes in the PV, V, and PM time-course), and that in addition passed the fold-change and FPKM filters described above, were kept as the final set of differentially expressed genes (DEGs). Gene models from the 12X.CRIBI-V1 grapevine reference annotations version were used, while the corresponding gene function and ID in version 12X.VCost.v3 was updated according to the grapevine gene reference catalogue (Grimplet *et al.*, 2012; Navarro-Payá *et al.*, 2021).

#### Clustering and functional analysis of differentially expressed genes

To determine whether DEGs between the white- and black-berried somatic variants that were detected in each of the four berry ripening series (TS, TF, GS and GF) were also differentially expressed in the other three series, the IntercatiVenn web tool was used to create a Venn diagram (Heberle *et al.*, 2015). After log_10_-transformation of the FPKM means, *z*-scores were calculated in order to standardize the expressions of the DEGs for each of the four series. The optimal number of clusters on each of the four ripening series was estimated by hierarchical clustering analysis using the agglomeration method ‘ward.D2’ implemented in the ‘hclust’ function of the stats R package. The final number of clusters was identified by a tree threshold cut-off in this preliminary hierarchical clustering (see Supplementary Fig. S1). The K-means algorithm of pheatmap v.1.0.12 (https://CRAN.R-project.org/package=pheatmap) was then applied to reach the estimated number of clusters. For genes grouped on each cluster of DEGs, Gene Ontology (GO) functional enrichment analysis was performed with ShinyGO 0.76 (Ge *et al.*, 2020), using the CRIBI grapevine gene IDs implemented in the tool, and considering FDR<0.05 and two genes of minimum pathway size for significant enrichment.

### Metabolite composition analysis

#### Targeted metabolite quantification

We carried out targeted quantification of primary (sugars, organic acids, and amino acids) and secondary metabolites (anthocyanins and flavonols). The skin and flesh were separated from 10–15 thawed berries per sample as described for RNA extraction. The skin samples were lyophilized using a VirTis Benchtop K (SP Scientific, Philadelphia, PA, USA) and then homogenized in a bead mill (MM200, Retsch). The flesh samples were squeezed inside plastic bags to obtain juice.

From the lyophilized powdered skin, 80 mg and 20 mg samples were used for extraction of primary and secondary metabolites, respectively. The flesh juice was centrifuged at 4500 *g* for 10 min (Eppendorf 5804, rotor A-4-44) and the supernatant was used for extraction of primary metabolites. Extractions and quantifications were performed as described by Torres *et al.* (2017). In brief, glucose, fructose, and malic acid were determined using an enzyme-coupled spectrophotometric method. Tartaric acid was determined using a colorimetric method based on ammonium vanadate reactions (Pereira *et al.*, 2006). A Bran and Luebbe TRAACS 800 AutoAnalyzer (Bran & Luebbe, Plaisir, France) was used for sugar and organic acid quantification. Amino acids were derivatized according to Hilbert *et al.* (2003) and analysed in a UHPLC UltiMate 3000 equipped with an FLD-3000 Fluorescence Detector (both Thermo Scientific). All proteinogenic (except methionine and tryptophan) as well as γ-aminobutyric acid (GABA) amino acids were quantified. Skin anthocyanins and flavanols were extracted in methanol with 10% hydrochloric acid and analysed in an UltiMate 3000 UHPLC equipped with a DAD-3000 (Thermo Scientific). Flavonoid identification was carried out by MS and NMR spectrometry (MS-NRMS) (Acevedo De la Cruz *et al.*, 2012; Hilbert *et al.*, 2015).

#### Untargeted volatile and precursor compound metabolomics

Aroma precursor and volatile compounds were quantified as described by Rambla *et al.* (2016). The skin and flesh were separated from 12 frozen berries per sample and homogenized in liquid nitrogen. Volatiles were quantified from frozen material at the Instituto de Biología Molecular y Celular de Plantas (Valencia, Spain), whilst precursors were quantified from lyophilized frozen powder at the Casaccia Research Centre (Rome, Italy). For volatile compounds, 2.2 g of CaCl_2_.2H_2_O and 1 ml of 100 mM EDTA were added to 1 g of frozen homogenized tissue, followed by headspace solid-phase microextraction and separation, and then determination by GC coupled to MS (HS-SPME/GC-MS). Chromatography was performed on an Agilent Technologies 6890N Gas Chromatograph, and results were recorded and processed using the Agilent Enhanced ChemStation software. The content of aroma precursors was evaluated from 50 mg lyophilized tissue. Non-polar precursors (carotenoids and chlorophylls) were analysed and quantified by LC/diode array detector/atmospheric pressure chemical ionization/high-resolution MS (LC-DAD-APCI-HRMS). Semi-polar precursors (terpene glucosides, amino acids, and phenylpropanoids) were analysed by LC/electrospray ionization/high-resolution MS [LC-ESI(+)-HRMS] analysis. LC-MS analyses were carried out using an LTQ-Orbitrap Discovery MS system operating in positive ESI, coupled to an Accela U-HPLC system (both ThermoFisher Scientific).

#### Phytohormone quantification

Phytohormone content was analysed from 100 mg of frozen homogenized skin obtained from seven berries per replicate. The analysis was conducted at the Centro de Edafología y Biología Aplicada del Segura (CEBAS) facilities through UHPLC-MS as described previously (Albacete *et al.*, 2008; Großkinsky *et al.*, 2014). Phytohormone analysis included the ethylene precursor 1-aminocyclopropane-1-carboxylic acid (ACC), cytokinins (*trans*-zeatin, t-Z; isopentenyladenine, iP; and zeatin riboside, ZR), gibberellins (GA_1_, GA_3_, GA_4_), auxin indoleacetic acid (IAA), abscisic acid (ABA), jasmonic acid (JA), salicylic acid (SA), and brassinosteroids (epibrassinolide, EB; 6-deoxo-24-epicastasterone, EC).

#### Statistical analysis of metabolite composition

One-way ANOVA and Tukey’s HSD post-hoc test were conducted in SPSS Statistics v. 26 to identify significant differences among the four accessions (TT, TB, GT, GB). Principal component analysis (PCA) and partial least-squares discriminant analysis (PLS-DA) multivariate statistical analyses were conducted using XLSTAT 2018 (Addinsoft). To identify metabolites with significant contributions to the PLS-DA discrimination of samples by grape colour, a threshold of variable importance in projection (VIP >1) was applied to the PLS-DA results.

## Results

### Grape-colour somatic variants display comparable technological ripening

**Fig. 1.**
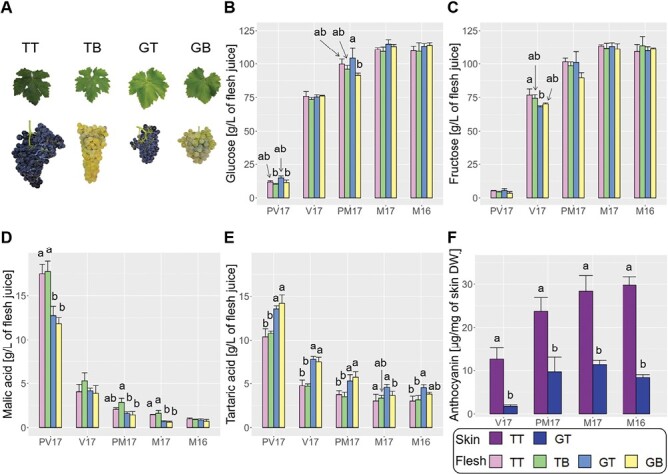
Accumulation of ripening-indicative metabolites in grape-colour somatic variants. (A) Representative images of grape bunches and leaves at harvest of the black-skinned berry cultivar Tempranillo Tinto (TT) and its white-skinned berry somatic variant Tempranillo Blanco (TB), and of black-skinned Garnacha Tinta (GT) and its white-skinned somatic variant Garnacha Blanca (GB). (B–E) Concentrations of metabolites in the juice of berry flesh at different development stages: (B) glucose, (C) fructose, (D) malic acid, and (E) tartaric acid.(F) Anthocyanin concentration in the skin of the black-skinned cultivars. Results are shown for the developmental stages pre-veraison (PV17), veraison (V17), and maturity (M17) for the 2017 season together with the maturity stage (M16) from 2016 season. Data are means (±SD), *n*=3. At each stage, different letters indicate significant differences among means as determined using ANOVA followed by Tukey’s HSD post-hoc test (*P*<0.05).

As a first approach to determining possible effects of grape colour variation on technological ripening (characterized by the sugar content and the titratable acidity of the grape must), we compared the contents of primary metabolites between the white-berried TB and GB and their respective black-berried TT and GT somatic variants (Fig. 1A). As expected given that the berries were selected for the same density at each post-veraison sampling stage to avoid displacements in phenology, there was no relevant variation in the concentrations of glucose and fructose in the berry flesh juice and a similar lack of differences was found in pre-veraison berries selected according to their green colour and hard touch (Fig. 1B, C). Whilst there were varietal differences in malic and tartaric acid in the flesh juice (Fig. 1D, E), the lack of differences between the berry-colour somatic variants of the same variety indicated that they had a similar berry-ripening physiology, and thus that our experimental system was appropriate. Similar patterns for sugars and organic acids were observed in the berry skins (Supplementary Table S2). In terms of grape colour, there were varietal differences for the content of anthocyanins in the fruit skin of the black-berried accessions, with concentrations in TT being more than double those in GT (Fig. 1F). The similar global trends observed at maturity (M) in 2016 and 2017 supported the reproducibility of the experiments, at least for the final outcome at harvest.

### Grape-skin anthocyanins increase berry temperature under exposure to solar radiation

**Fig. 2.**
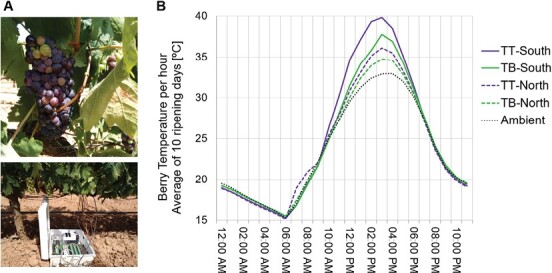
Daytime temperature of berries is lower in grape somatic variants that lack anthocyanins. (A) Experimental set-up with thermocouples connected to ripening berries. (B) Mean hourly berry temperature determined over a period of 10 sunny days during the mid-ripening stage (16–26 August, 2016) in Tempranillo Blanco (TB) and Tempranillo Tinto (TT) berries that were on either the south- or north-facing side of vineyard rows. The ambient temperature was recorded at the canopy level. Mean temperatures were determined from measurements taken every 30 s. along with the ambient air temperature in the same TT and period. The results are consistent with measurements recorded at earlier and later stages of ripening under more variable environmental conditions (Supplementary Fig. S2).

To examine possible effects of the presence/absence of anthocyanins on the internal tissue microenvironment of the berries, we measured the temperature of ripening berries in the Tempranillo somatic variants using thermocouples (Fig. 2A). The temperature of ripening berries was always higher than the ambient temperature during the daytime but the difference disappeared during the night (Fig. 2B; Supplementary Table S3). The mean berry temperatures recorded during a period of 10 consecutive sunny days at mid-ripening were as much 3.5 ºC lower in the white- than in the black-berried Tempranillo at solar noon in south-facing row side of the vineyard. A similar pattern was observed in berries from the north-facing side, although the greatest temperature differences between the berry-colour somatic variants were lower and were shifted toward earlier times of day. Temperature differences were similarly higher in TB than in TT during an earlier 4 d period post-veraison and also to a slightly lesser extent during a later 29 d period pre-harvest that included some cloudy and rainy days (Supplementary Fig. S2). These results indicated that the light absorbed by anthocyanins in the berry skin led to increased berry temperature, and thus to different internal tissue microenvironments between the black and white ripening grapes.

### RNA-seq transcriptome analysis of effects associated with berry colour

**Fig. 3.**
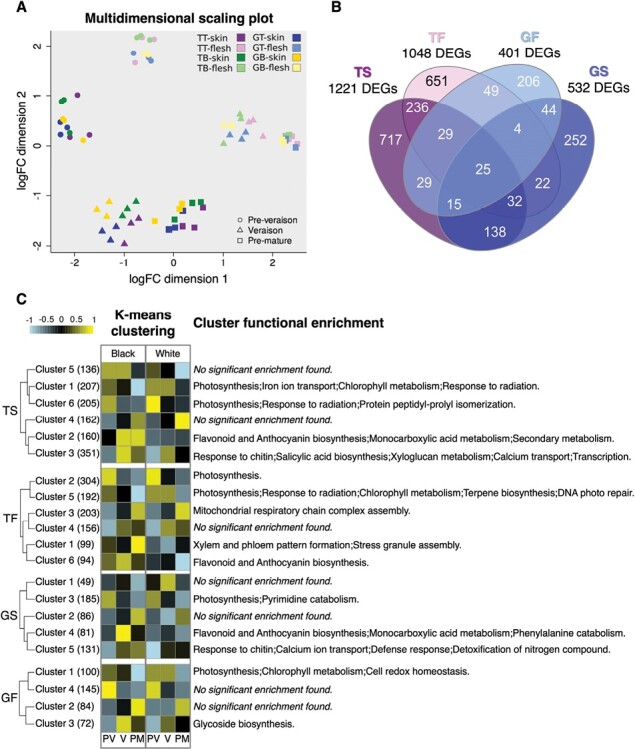
Variation in grape skin colour alters the berry transcriptome in processes beyond those related to anthocyanin biosynthesis. (A) Multidimensional scaling plot of two dimensions depicting the variability of gene expression among all samples used for RNA-sequencing in different berry tissues and at different developmental stages in 2016. TT, Tempranillo Tinto; TB, Tempranillo Blanco; GT, Garnacha Tinta; GB, Garnacha Blanca. (B) Venn diagram showing the relationship among differentially expressed genes (DEGs) identified by RNA-seq in the four ripening series: TS, Tempranillo berry skin; TF, Tempranillo berry flesh; GS, Garnacha berry skin; and GF, Garnacha berry flesh. (C) Clustering of DEGs in each of the four ripening series and functional enrichment analysis. PV, pre-veraison (green); V, veraison; PM, pre-maturity. DEGs were identified according to a 5% false-discovery rate using maSigPro in a two-class time series (white- versus black-berried variants compared along the series) and a ≥1.75-fold change in at least one stage. The optimum cluster number in each series was determined by a tree threshold cut-off in a preliminary hierarchical clustering analysis (Supplementary Fig. S1). The heatmaps show the normalized *z*-score FPKM expression values averaged across gene clusters. A summary of the non-redundant over-represented functional categories is shown; results from the full functional enrichment analysis are shown in Supplementary Table S5.

We conducted an RNA-seq experiment comparing white- to black-berried somatic variants during the berry development and ripening series of PV, V, and PM stages in the 2016 season to study effects on gene expression, although it should be noted that not all differences might be translated through to active protein expression. We analysed the berry skin and flesh separately to look for grape colour effects that could be direct in the skin where MYBA1-MYBA2 functioning primarily acts, or indirect if they also affect the flesh. An MDS plot from the RNA-seq dataset identified ripening stage and tissue as the factors most contributing to transcriptome variation (Fig. 3A). Berry colour and cultivar effects were also evident, mostly in the skin from the veraison stage.

We then specifically searched for DEGs between the white- and black-berried somatic variants of the same cultivar along the development and ripening time-course series for each tissue. A greater number of colour-associated DEGs was identified in Tempranillo than in Garnacha and in berry skin than in flesh, with 1221 being detected in the white- versus black-berried variant time-course series of Tempranillo skin (TS), 1048 in Tempranillo flesh (TF), 532 in Garnacha skin (GS), and 401 Garnacha flesh (GF) (Fig. 3B; Supplementary Table S4).

#### Absence of berry-skin anthocyanins is associated with higher expression of photosynthesis-related and light-responsive genes

We next performed clustering analysis of the DEGs detected for each of the four ripening series and functional enrichment analysis on each of the resulting cluster groups. In all four series, the most frequent pattern involved DEGs with higher expression in the white- than in the black-berried variants from veraison that were down-regulation as ripening progressed (Fig. 3C; Supplementary Table S4; clusters TS 1, TS 6, TF 2, TF 5, GS 3, and GS 1), and these were generally over-represented in ‘Photosynthesis’ and related GO terms such as ‘Chlorophyll metabolism’ (Supplementary Table S5). There was over-representation of ‘DNA photoreactive repair’ in cluster TF 5 (genes down-regulated during ripening in the flesh but with higher expression in TB than TT from the V stage and no difference in PV stage), and this cluster was also over-represented in ‘Terpene biosynthetic process’, indicating that radiation-related functions were up-regulated in the flesh of TB compared to TT upon anthocyanin accumulation in the latter.

#### Specific activation of the phenylpropanoid pathway and flavonol trihydroxylation in anthocyanin-pigmented grapes

A group of genes up-regulated upon ripening and showing higher expression in the black-berried somatic variants was also prominent in the clustering analysis. This included clusters TS 2, TF 6, GS 4, and GF 3, comprising DEGs with great up-regulation from the veraison stage in the black-berried variants and steady low expression in the white-berried variants in each of the four ripening series (Fig. 3C; Supplementary Table S5). *VviMYBA1* (Vitvi02g01019 = VIT_02s0033g00410) was present in these clusters, and was therefore induced in the skin of the black-berried variants as expected, and it although the absolute expression levels (in terms of RNA-seq read counts) were much lower in the flesh, they showed the same pattern (Supplementary Fig. S3; Supplementary Table S4). The skin series clusters TS 2 and GS 4 as well as the flesh TF 6 cluster were indeed over-represented in genes classified in the phenylpropanoid biosynthesis-related categories ‘Flavonoid biosynthetic process’ and ‘Anthocyanin-containing compound biosynthetic process’ (Fig. 3C; Supplementary Table S5). Similarly, cluster GF 3 was over-represented in ‘Glycoside biosynthetic process’ category, in which the gene encoding UFGT participates (*VviUFGT*, Vitvi16g00156 = VIT_16s0039g02230).

**Fig. 4.**
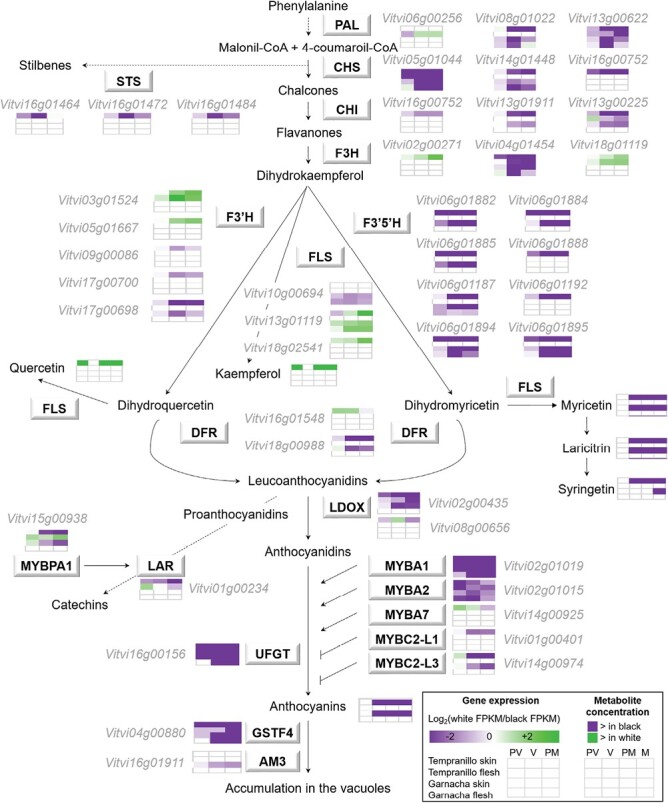
Activation of the phenylpropanoid pathway and of flavonol trihydroxylation in the presence of functional *VviMYBA1* and *VviMYBA2* genes in different grape-colour somatic variants. The diagram shows the phenylpropanoid and flavonoid biosynthesis pathway and the regulatory MYB transcription factors. The differentially expressed genes (DEGs) identified by RNA-seq are displayed in boxes (Supplementary Table S4). The heatmaps show significant differences in DEG expression ratios (white/black) in the skin and flesh of the Tempranillo and Garnacha cultivars at the pre-veraison (PV), veraison (V), and pre-maturity (PM) stages, as indicated in the key. Significant differences for metabolite contents were determined by Student’s *t*-test (*P*<0.05) or VIP>1 for colour factor PLS-DA. In addition, berries at maturity (M) were also analysed for metabolite content. Pathway metabolite names are displayed without boxes. PAL, phenylalanine ammonia-lyase; CHS, chalcone synthase; CHI, chalcone isomerase; STS, stilbene synthase; F3´H, flavonoid 3´ hydroxylase; F3´5´H, flavonoid 3´5´ hydroxylase; FLS, flavonol synthase; DFR, dihydroflavonol 4-reductase; LDOX, leucoanthocyanidin dioxygenase; UFGT, UDP-glucose:flavonoid 3-O-glucosyltransferase.

Inspection of the DEGs in the phenylpropanoid pathway (Fig. 4; Supplementary Fig. S3; Supplementary Table S4) identified genes with expression patterns paralleling those of anthocyanin accumulation (reduced expression in white- compared to black-berried variants in the skin because of specific up-regulation from the V stage in black-berried variants), including one phenylalanine ammonia-lyase (*PAL*, Vitvi08g01022 = VIT_08s0040g01710), two chalcone isomerases (*CHI*, Vitvi16g00752 = VIT_16s0022g01020; and Vitvi13g01911 = VIT_13s0067g02870), one flavonoid 3´ hydroxylase (*F3´H*, Vitvi17g00698 = VIT_17s0000g07200), one dihydroflavonol 4-reductase (*DFR*, Vitvi18g00988 = VIT_18s0001g12800), one leucoanthocyanidin dioxygenase (*LDOX*, Vitvi02g00435 = VIT_02s0025g04720), and six flavonoid 3´5´ hydroxylase (*F3*´*5*´*H*; Vitvi06g01882 = VIT_06s0009g02810; Vitvi06g01884 = VIT_06s0009g02830; Vitvi06g01885 = VIT_06s0009g02840; Vitvi06g01187 = VIT_06s0009g02920 and VIT_06s0009g02910; Vitvi06g01894 = VIT_06s0009g03000; and Vitvi06g01895 = VIT_06s0009g03010). Increased activity of F3´5´H enzymes would not only result in trihydoxylated anthocyanins in the black berries, but also in the trihydroxylation of colourless phenylpropanoids such as flavonols irrespective of berry colour (Bogs *et al.*, 2006). Quantification of berry skin flavonols indeed confirmed that myricetin, the major trihydroxylated flavonol, accumulated from the V stage exclusively in the black-berried variants (Fig. 4; Supplementary Fig. S4A). Much lower levels of the trihydroxylated flavonols laricitrin and syringetin were also detected in white- than in black-berried variants (Fig. 4; Supplementary Fig. S4B, C).

#### Enhanced activation of pathogen-defence gene expression with ripening in the skin of anthocyanin-pigmented grapes

Functional categories related to pathogen defence and signalling, such as ‘Defense response’, ‘Response to chitin’, ‘Calcium ion transmembrane transport’, and ‘Salicylic acid (SA) biosynthetic process’, were over-represented in the TS 3 and GS 5 clusters of genes that were up-regulated upon ripening in the skin, and had higher peaks of expression in the black-berried variants (Fig. 3C; Supplementary Table S5). These clusters in both Tempranillo and Garnacha included several nucleotide-binding leucine-rich repeat (NLR) genes, and chitin- and pathogen-response genes, as well as a homolog (Vitvi03g00033 = VIT_03s0038g00430) of Arabidopsis *ENHANCED DISEASE SUSCEPTIBILITY 5* (*EDS5*)/*SALICYLIC ACID INDUCTION DEFICIENT 1* (*SID1*), which is a transporter essential for SA accumulation (Serrano *et al.*, 2013; Supplementary Table S4).

To test for their possible contributions to the activation of pathogen defence responses in the black-berried cultivars, we quantified phytohormones in the skins of berries at the PM stage. To assess possible direct relationships between hormone content and the differential expression in pathogen signalling and defence genes that we observed, we decided to quantify phytohormone levels in the same 2016 pre-maturity berry skin samples used in the RNA-seq (Supplementary Table S1). SA content was higher in Tempranillo than in Garnacha irrespective of somatic variation for berry colour (Table 1). JA, which is also involved in biotic stress responses, was significantly lower in GB compared to GT. On the other hand, the accumulation of ABA, which is associated with abiotic stress responses and with grape ripening, was significantly higher in TB than in TT. There were no significant colour-associated effects for the other phytohormones analysed (Supplementary Table S6). The differences in JA together with the related transcriptome changes indicated possible direct or indirect roles of *VviMYBA1*-*VviMYBA2* as triggers of protection against pathogens.

**Table 1.** Variation in berry skin phytohormone content between grape-colour somatic variants

Hormone	Phytohormone content (ng g^−1^ skin DW)				ANOVA *P*-value
	TT	TB	GT	GB	
ABA	134.7 ± 29.6^a^	358.8 ± 34.1^b^	271.5 ± 40.8^b^	348.9 ± 45.4^b^	<0.001
JA	16.1 ± 1.7^a^	15.3 ± 2.4^a^	13.0 ± 1.2^a^	7.3 ± 0.3^b^	0.001
SA	65.9 ± 29.9^a^	59.6 ± 13.2^a^	17.5 ± 7.2^b^	16.4 ± 2.4^b^	0.01

ABA, abscisic acid; JA, jasmonic acid; SA, salicylic acid. TT, Tempranillo Tinto; TB, Tempranillo Blanco; GT, Garnacha Tinta; GB, Garnacha Blanca. Data are means ±SD, *n*=3. Additional results for other hormones are given in Supplementary Table S6. Different letters indicate significant differences among means as determined using ANOVA followed by Tukey’s HSD post-hoc test (*P*<0.05).

#### Gene dosage as a side-effect of deletions causing somatic variation in grape colour

For the DEGs that were located in the genome regions in which TB has been reported to be hemizygous in chromosomes 2 and 5 (which comprise 313 genes in total; Carbonell-Bejerano *et al.*, 2017), 86 out of 87 and 90 out of 94 in the TS and TF berry development series, respectively, were down-regulated in TB compared to TT irrespectively of the ripening stage (Supplementary Fig. S5; Supplementary Table S4). The expression of most of these genes was unaffected in GB compared to GT, in agreement with the much shorter berry colour loss-causing deletion estimated for the GB accession, which is hemizygous only for 35 genes around the grape-colour locus in LG 2 (Royo *et al.*, 2019), and there were expression differences affecting *VviMYBA1* and *VviMYBA2* (Supplementary Fig. S5; Supplementary Table S4). Taken together, these differences indicated gene dosage effects reducing expression in monosomic genome regions left after the somatic deletions. Decreased gene dosage and unmasking of deleterious recessive alleles due to hemizygosity, together with impaired meiosis, could collectively underlie the pleiotropic phenotypes related to berry colour and gamete viability reported in TB (Carbonell-Bejerano *et al.*, 2017; Tello *et al.*, 2021; Kizildeniz *et al.*, 2022). Remarkably, the DEGs identified in any of the cultivars and tissues overlapping with the specific hemizygous intervals of TB compared to TT did not include any known regulator of metabolism pathways, apart from the phenylpropanoid regulators *VviMYBA1* and *VviMYBA2* (Vitvi02g01015), and the pseudogene *VvMYBA8* (Vitvi02g01310) resulting from the frameshift InDel in *VviMYBA2* (Supplementary Table S4).

### Effects on accumulation of aroma-related compounds associated with berry colour variation between somatic variants

Because the transcriptomic results indicated that there were possible effects of grape colour on processes contributing to grape and wine aroma, we quantified volatiles and precursors in mature grapes collected in the 2016 season. Given that most differences in gene expression between the white- and black-berried somatic variants were established form the V stage (Fig. 3; Supplementary Table S4), and under the assumption that variations in the grape composition at harvest should represent the cumulative effect of all gene expression changes observed during berry ripening in the RNA-seq samples collected up to 6 September 2016, we quantified volatile compounds and aroma precursors at maturity (13 September, 2016), with the intention of assessing the impact of berry colour on the organoleptic properties of the final product that would be used at harvest time for winemaking or fresh consumption of the fruit (Supplementary Table S1). A total of 124 different metabolites were detected from non-polar, semi-polar, and volatile compounds (Supplementary Table S7). Both PCA and PLS-DA identified clear effects of grape colour variation, and these were more pronounced for non-polar precursors than for semi-polar precursors or volatiles (Supplementary Fig. S6). While variation was higher in the berry skin, differences were also evident in the flesh, indicating for effects in aroma precursor accumulation that were not directly related to the presence or the metabolism of anthocyanins (Supplementary Fig. S6B–D, F). In most cases the PLS-DA showed more extreme colour effects in Tempranillo than in Garnacha, but we mostly focus below on differences that were consistent between the white- and black-berried somatic variants in both cultivars, and hence most likely result from the loss of function of *VviMYBA1* and *VviMYBA2* that is the only major-effect genome variation shared by the TB and GB white-berried somatic mutant accessions in our study (Carbonell-Bejerano *et al*., 2017; Royo *et al*., 2019).

#### Effects on non-polar precursors

**Fig. 5.**
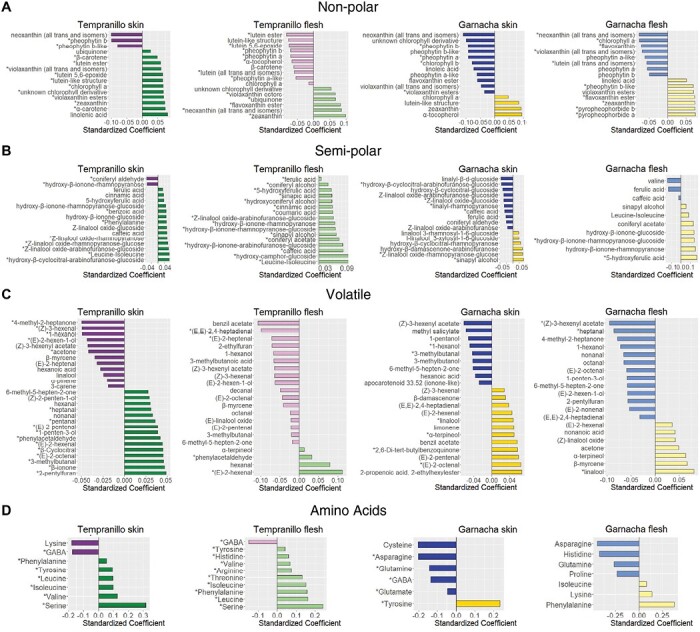
Effects of variation in berry skin colour on the contents of aroma precursors and volatile compounds in different grape-colour somatic variants. Standardized coefficients are shown for compounds with VIP>1 in the PLS-DA for berry-colour factor carried out on berry skin and flesh samples. The full table of metabolite content and PLS-DA results is given in Supplementary Table S7. (A–C) Berries at maturity collected in the 2016 season and compounds were analysed by PLS-DA. (A) Non-polar compounds, (B) semi-polar aroma precursors, and (C) volatile compounds. (D) Amino acids were analysed by PLS-DA collectively from pre-maturity and maturity berry stages from the 2016 season. For each cultivar genetic background (Tempranillo or Garnacha), positive and negative PLS-DA coefficients respectively indicate higher and lower metabolite accumulation in the white-berried somatic variant compared to the corresponding black-berried one. Asterisks indicate that the difference between the white- and black-berried variants is significant, as determined by Student’s *t*-test (*P*<0.05).

PLS-DA identified the carotenoid zeaxanthin as a significant compound contributing to the grape colour factor (Fig. 5A). Accumulation of zeaxanthin was indeed significantly greater in white- than in black-berried variants in all cases according to *t*-test comparisons, with a mean fold-difference in content of >3.1 in the skin tissue, in which relative accumulation levels were higher than in the flesh (Supplementary Table S7). A carotenoid with a lutein-like structure also showed significantly higher accumulation in the white variants for both cultivars, but only in the berry skin tissue. Similarly, chlorophyll *a* was higher in the skin of the white variants, although the difference was only significant in Tempranillo and the pattern was the opposite in the berry flesh. On the other hand, PLS-DA showed that the white variants accumulated significantly lower levels of the pheophytin *b* and pheophorbide *b*-like chlorophyll breakdown products as well as of the carotenoid neoxanthin in the skin for both cultivars (Fig. 5A). In addition, significantly increased contents in *β*-carotene and free and esterified violaxanthin were observed in the white-berry skin of TB compared to TT, which might have been related to the increased ABA levels (Supplementary Table S6).

#### Effects on semi-polar precursors

**Fig. 6.**
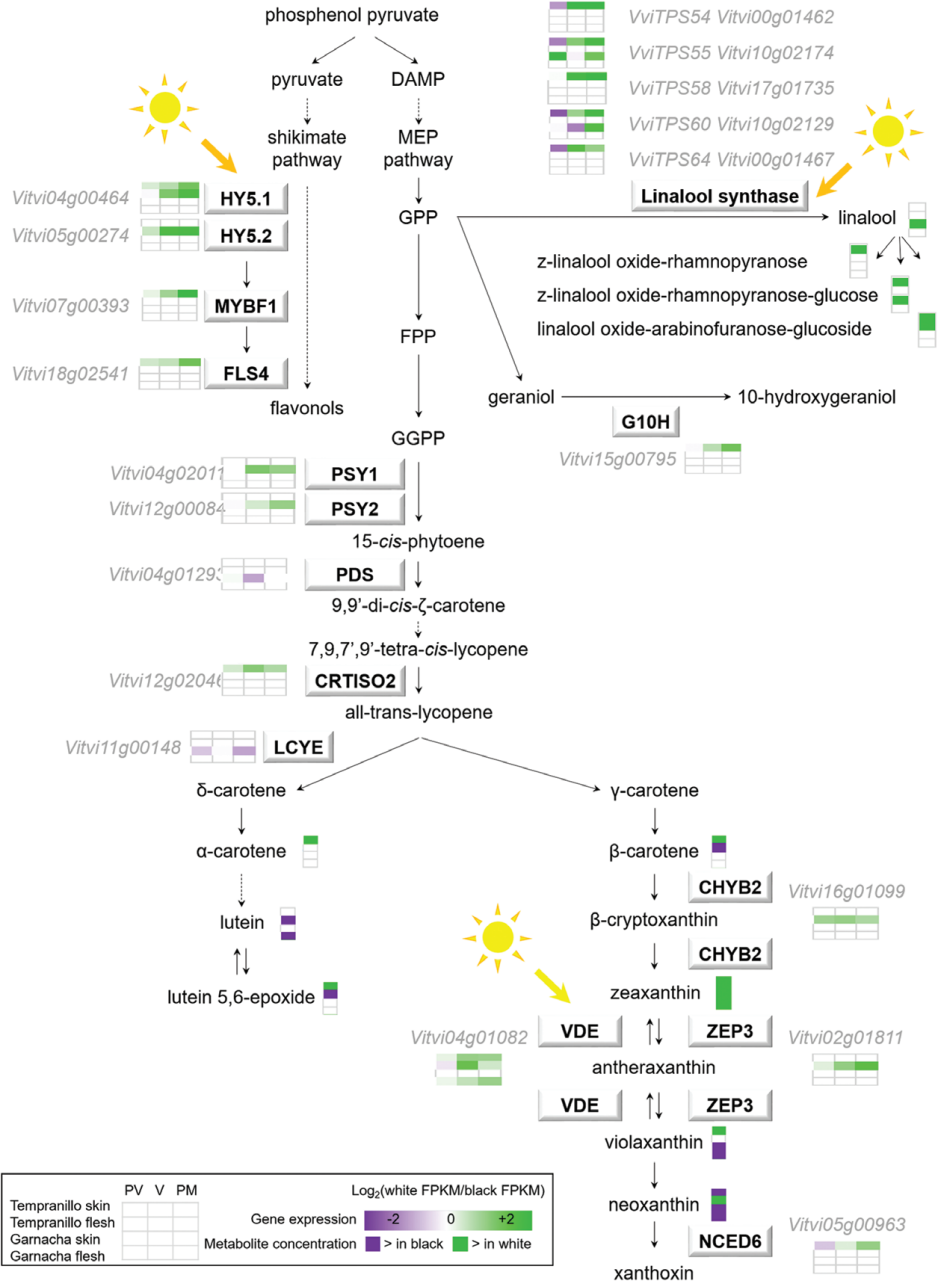
Activation of light-responsive flavonol and terpene metabolism in white berries. The diagram shows the terpene-mevalonate (MEV) and shikimate-flavonol biosynthetic pathways, together with light-signalling genes. The differentially expressed genes (DEGs) identified by RNA-sequencing are displayed in boxes (Supplementary Table S4). The heatmaps show significant differences in DEG expression ratios (white/black) in the skin and flesh of the Tempranillo and Garnacha cultivars at the pre-veraison (PV), veraison (V), and pre-maturity (PM) stages, as indicated in the key. Significant differences for metabolite contents were determined by Student’s *t*-test (*P*<0.05) or VIP>1 for colour factor PLS-DA. In addition, berries at maturity (M) were also analysed for metabolite content. Pathway metabolite names are displayed without boxes. FLS, flavonol synthase; G10H, geraniol 10-hydroxylase; PSY1-2, phytoene synthase 1 and 2; PDS, phytoene desaturase; CRTISO2, carotenoid isomerase 2; LCYE, lycopene ξ-cyclase; CHYB, β-carotene hydroxylase; VDE, violaxanthin de-epoxidase; ZEP3, zeaxanthin epoxidase 3; CHYB2, β-carotene hydroxylase 2; NCED6, 9-cis-epoxycarotenoid dioxygenase 6; DMAP, dimethylallyl phosphate; GPP, geranyl pyrophosphate; FPP, farnesyl pyrophosphate; GGPP, geranylgeranyl pyrophosphate.

The glycosylated monoterpene (Z)-linalool oxide-rhamnopyranose-glucose accumulated at significantly higher levels in the white variants for all tissues and cultivars, with a mean fold-difference of >2.7 in the skin tissue, where the relative level of accumulation was higher than in the flesh (Figs 5B, 6; Supplementary Table S7). The glycosylated norisoprenoid hydroxy-β-ionone-rhamnopyranose-glucoside also showed higher accumulation in the white-berried variants, although differences were only significant in the flesh. Other glycosylated monoterpenes, including several linalool forms, accumulated to higher levels in the skin of TB compared to TT, but showed the opposite between GB and GT. In terms of phenylpropanoid metabolism, the accumulation of the hydroxycinnamic acid 5-hydroxyferulic acid was also significantly higher in the white variants in the berry skin. In addition, benzoic acid as well as the amino acids leucine/isoleucine and phenylalanine were detected at significantly higher levels in TB than in TT in the skin. Coniferyl aldehyde levels were consistently higher in black- compared to white-berried variants in the skin (Fig. 5B; Supplementary Table S7).

#### Effects on volatiles

The accumulation of the free monoterpenes linalool and α-terpineol was higher in the GB pericarp than in GT according to PLS-DA, with significant differences for linalool in both the tissues, but in the skin only for α-terpineol (Fig. 5C; Supplementary Table S7). The content of the C13-norisoprenoid β-ionone was significantly higher in the skin of TB compared to TT, and the same trend without significant differences was observed in the skin of Garnacha (Supplementary Table S7).

The levels of the fatty acid-derived aldehydes (E)-2-octenal and (E)-2-pentenal were significantly higher in the white-berried variants for both cultivars in the skin tissue. The same was observed for the C6-derived aldehyde (E)-2-hexenal, which accumulated at higher levels in the white-berried variants in both tissues, although the differences were only significant in Tempranillo, with >1.5-fold difference (Fig. 5C; Supplementary Table S7).

Among the free volatiles that accumulated to a higher extent in the black- compared to white-berried variants, the C6-derived alcohol 1-hexanol and the monocarboxylic acid ester (Z)-3-hexenyl acetate showed >3.7-fold and significant differences in all cases (Fig. 5C; Supplementary Table S7). The same trend was observed for another C6-derived alcohol, (E)-2-hexen-1-ol, with >6.8-fold differences, although the difference was only significant in the skin of Tempranillo.

#### Effects on amino acids

Given the effects for phenylpropanoid precursor amino acids (e.g. phenylalanine) that we observed from the metabolomics of the semi-polar precursors, and also considering that grape amino acids play a role as aroma precursors upon wine fermentation (Fairbairn *et al.*, 2017), we broadly targeted quantification of amino acids at the harvest stage in the 2016 season, and during grape ripening in the 2017 season. PCA and PLS-DA showed that differences between the grape-colour somatic variants mostly occurred at the PM and M stages in both the berry tissues (Supplementary Fig. S7, Supplementary Table S2). Focusing on these two stages, our analysis confirmed that there was higher accumulation of phenylpropanoid precursor amino acids in the white-berried variant samples, including higher phenylalanine content in all of them except in the GB skin, and higher tyrosine content in the skin of both the white-berried variants (Fig. 5D; Supplementary Table S2). Higher contents of branched-chain amino acids (BCAAs) were observed in the white-berried variants for valine, leucine, and isoleucine in TB compared to TT in both tissues, and for isoleucine in the flesh of GB compared to GT. On the other hand, the white-berried variants showed significantly lower concentrations of the non-proteinogenic amino acid GABA, in both pericarp tissues for TB and in the skin for GB.

## Discussion

**Fig. 7.**
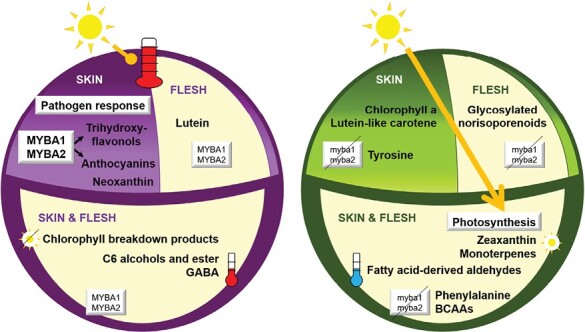
Proposed model for features of berries that are modulated by grape colour. Ripening anthocyanin-pigmented and white grapes are shown on the left and right, respectively. The features most likely associated with the functioning of MYBA1 and MYBA2 that were consistently identified between the white- and black-berried somatic variants of both Tempranillo and Garnacha cultivars are shown. The absence of anthocyanins in the white-berried variants leads to increased penetration of light within the berry (right), and a consequent up-regulation of genes in the ‘Photosynthesis’ functional category as well as to the accumulation of different light-responsive monoterpenes, carotenoids, norisoprenoids, and chlorophylls in the skin and flesh. The lack of anthocyanin production causes a higher accumulation of phenylalanine, tyrosine, and branched-chain amino acid (BCAAs) precursors in white grapes. In the skin of the black-berried variants (left), the function of MYBA1 and MYBA2 directly leads to increases in trihydroxyflavonols and anthocyanins, and is associated with an up-regulation of genes in the functional category ‘Pathogen response’. Reduced light penetrance due to absorption by anthocyanins in black grapes is associated with increased accumulation of neoxanthin and lutein in the skin and flesh, respectively, and of chlorophyll breakdown products in both the tissues. Absorption of light by anthocyanins increases the temperature of the pericarp of black grapes triggering a higher accumulation of γ-aminobutyric acid (GABA) and C6 alcohols and esters compared to white grapes that accumulate higher fatty acid-derived aldehydes associated to their lower temperature due to lower light absorption.

Several studies have highlighted differences in gene expression between white and anthocyanin-pigmented grapes in relation to phenylpropanoid metabolism as well as in light signalling and photosynthetic processes (Rinaldo *et al*., 2015; Massonnet *et al*., 2017; Ferreira *et al*., 2019; Zhang *et al*., 2021, Preprint). Adopting a systematic approach to characterize side-effects of grape-colour variation, we performed a joint microenvironment, transcriptomics, and metabolomics study that compared near-isogenic grape-colour somatic variants in the genetic backgrounds of two cultivars, and we standardized the ripening stage among the variants by sorting the berries according to density in all the post-veraison samples that were compared (Supplementary Table S1). The TB and GB white-berried somatic variants that we studied are hemizygous for 313 and 35 genes (1% and 0.11% of genes in the genome), respectively (Carbonell-Bejerano *et al.*, 2017; Royo *et al.*, 2019), and our RNA-seq results showed that this hemizygosity had gene-dosage consequences that resulted in decreased expression levels for 180 hemizygous genes in TB green and/or ripening berries (Supplementary Fig. S5; Supplementary Table S4), which might have additional effects on grape composition; nevertheless, only *VviMYBA1* and *VviMYBA2* were DEGs and/or involved loss-of-function mutations in both TB and GB compared to their black-berried counterparts. Therefore, effects on berry gene expression and composition that were shared between the GB versus GT and TB versus TT somatic variants are expected to be consequence only of the two loss-of-function genes due to the somatic genome rearrangements that they share, which involve *VviMYBA1* and *VviMYBA2* at the grape-colour locus (Carbonell-Bejerano *et al.*, 2017; Royo *et al.*, 2019). These shared effects revealed consequences of grape-colour variation on berry composition, and not only confirmed effects on phenylpropanoids and related pathways, but also demonstrated for the first time an impact on the accumulation of aroma precursors such as terpene and fatty acid-derived volatiles, or branched-chain amino acids, as well as on stress-signalling and other protective molecules such as GABA (Fig. 7). Our findings also reveal for the first time the possible mechanisms involved, including a higher temperature in anthocyanin-coloured ripening grapes compared with white ones (Figs 2, 7; Supplementary Fig. S2). The fact that the increase in temperature correlated with the level of incident sunlight (Fig. 2; Supplementary Fig. S2; Supplementary Table S3), suggests that heat is produced upon absorption of light by anthocyanins in the skin of coloured grapes, whereas sunlight would penetrate further in white grapes and trigger specific light responses (Fig. 7). While the thermocouple system that we used recorded the overall temperature of the pericarp, the heating that we observed might have initially been generated in the berry skin upon absorption of light by the anthocyanins there and then transmitted to the flesh. Similar heating phenomena and their physiological consequences have been reported for other dark particles/molecules that accumulate in the surfaces of other living organisms (Hirano *et al.*, 1995; Rogalla *et al.*, 2021). Our findings indicate that in addition to the effects on pathways that are directly controlled by the MYBA1 and MYBA2 transcription factors, ripening white grapes display specific physiological and potential aroma features due to variations in the berry microenvironment in the absence of anthocyanin accumulation.

### Enhanced accumulation of light-inducible terpenes, C_ 6_ compounds, and flavonols in white grapes could result from higher penetration of radiation

A prominent characteristic we found in the fruits of the white-berried somatic variants in both the Tempranillo and Garnacha cultivars was their lower down-regulation of photosynthesis-related genes throughout ripening, which paralleled the accumulation of anthocyanins in their black-berried counterparts. This effect could be independent of MYBA action or anthocyanin metabolism as it was detected in the berry flesh samples in addition to the skin (Fig. 3C; Supplementary Table S5). It has previously been reported that photosynthesis genes display higher expression in white- compared to red-berry genotypes, supporting it being a colour-associated feature in grapes (Massonnet *et al.*, 2017; Ferreira *et al.*, 2019). Transcriptomic activation of photosynthesis has been described in grape berries upon defoliation (du Plessis *et al.*, 2017; He *et al.*, 2020), and paralleling increasing radiation and temperature during daytime in the white-berried cultivar Verdejo but not in black-berried Tempranillo (Carbonell-Bejerano *et al.*, 2014b). We can therefore hypothesize that higher radiation penetration into anthocyaninless grapes up-regulates photosynthesis genes in the pericarp compared to coloured grapes, and this partially counteracts the repression of these genes that is a characteristic of the grape ripening process (Fig. 3C) (Lijavetzky *et al.*, 2012). This in turn apparently affects berry composition, including >2-fold higher levels of the major photosynthesis pigment chlorophyll *a* being observed in the skin of the white-berried variants of both the Tempranillo and Garnacha cultivars (Fig. 5A; Supplementary Table S7), concurrent with their higher expression of chlorophyll biosynthesis genes (Fig. 3C; Supplementary Tables S4, S5). While mature grapes show chlorophyll fluorescence features indicating for photosynthetic activity that increases with incident light intensity (Garrido *et al.*, 2018), there is evidence to suggest that photosynthesis in mature fruits would not contribute to net assimilation of carbon in grapes and other fruits such as tomato (*Solanum lycopersicum*) (Cocaliadis *et al.*, 2014; Teixeira *et al.*, 2022). Nevertheless, we found that *GOLDEN2-LIKE1* (*GLK1*, Vitvi12g00260 = VIT_12s0028g03100) was up-regulated in the skin of TB compared to TT (Supplementary Table S4), which might contribute to higher chloroplast biogenesis and accumulation of carotenoids and sugars in the skin of white grapes, given the role of a radiation-responsive *GLK* homolog in tomato (Powell *et al.*, 2012; Nguyen *et al.*, 2014; Li *et al.*, 2018).

Supporting a higher penetration of sunlight radiation in white grapes, the UV-B light signalling transcription factors *VviHY5-1* (Vitvi04g00464 = VIT_04s0008g05210) and *VviHY5-2* (Vitvi05g00274 = VIT_05s0020g01090) and their direct targets *VviMYBF1* (Vitvi07g00393 = VIT_07s0005g01210) and *VviFLS4* (Vitvi18g02541 = VIT_18s0001g03470), which are involved in the biosynthesis of sunscreen flavonols (Carbonell-Bejerano *et al.*, 2014a; Loyola *et al.*, 2016), were up-regulated in TB compared to TT in both the skin and flesh tissues (Fig. 6; Supplementary Table S4). This could have been responsible for the tendency towards higher accumulation of dihydroxylated quercetin and monohydroxylated kaempferol flavonols in the skin of the white-berried variants (Supplementary Fig. S4), as has been described in the white-skin sectors of the black–white variegated grape cultivar ‘Béquignol Noir’ (Zhang *et al*., 2021, Preprint).

Dissipation of excessive light in exposed grape berries via tetraterpenoid carotenoids through the xanthophyll cycle, in which violaxanthin is de-epoxidized to zeaxanthin coinciding with the overexpression of a gene encoding a violaxanthin de-epoxidase (*VDE*, Vitvi04g01082 = VIT_04s0043g01010), has been described previously (Young *et al.*, 2016; du Plessis *et al.*, 2017). The higher accumulation of zeaxanthin in the white-berried variants of both Tempranillo and Garnacha correlated with their higher expression of *VDE* (Fig. 6; Supplementary Table S7), indicating not only that this cycle is activated by light as previously reported, but also that it is more active in white than in black ripening grapes. The accumulation of the monoterpene linalool is another major feature observed in light-exposed grapes (Sasaki *et al.*, 2016; Young *et al.*, 2016), and we found that two genes annotated as linalool synthase (*VviTPS55*, Vitvi10g02174 = VIT_00s0271g00010, and *VviTPS60*, Vitvi10g02129 = VIT_00s0385g00020) that are UV-B light-responsive (Martin *et al.*, 2010; Carbonell-Bejerano *et al.*, 2014a) were up-regulated in the skin of the two white-berried variants compared to their black-berried counterparts (Fig. 6; Supplementary Table S4). The highest up-regulation of *VviTPS55* and *VviTPS60* in the white-berried variants was detected at the PM stage, coinciding with the highest levels of anthocyanins in the black-berried variants (Fig. 1F; Supplementary Table S4). Additional linalool synthase paralogs were also up-regulated in TB. This differential gene expression seemed to alter grape composition as several glycosides of linalool oxide accumulated at higher levels in the white-berried variants, most notably in the skin of TB, as well as free linalool in the skin of GB (Fig. 6; Supplementary Table S7). Because linalool has a low perceptibility threshold and is sensed as floral and citrus notes (Wu *et al.*, 2016; Lin *et al.*, 2019), it could provide distinctive aroma features to white grapes and to the wines produced from them. Similarly, the higher accumulation of the carotene-derived C_13_-norisoprenoids hydroxy-β-ionone-rhamnopyranose-glucoside in the flesh of the white-berried variants and β-ionone in the skin of TB compared to TT (Fig. 5; Supplementary Table S7) could be light-inducible and contribute to flowery and fruity notes (Marais *et al.*, 1999; Liu *et al.*, 2015; Lin *et al.*, 2019). Indeed, QTLs for linalool, linalool oxides, and norsisoprenoids have consistently been identified before on LG2 (Doligez *et al.*, 2006b; Battilana *et al.*, 2009; Duchene *et al.*, 2009; Koyama *et al.*, 2022). Despite the fact that these QTLs co-localize with the grape-colour locus, they have not been related to variations in grape colour before. Our results demonstrate that the accumulation of specific aromatic terpenoids is increased in white grapes, suggesting that these QTLs might rely on the effects that variations in anthocyanin content determined by *VviMYBA* and *VviMYBA2* have in altering the berry microenvironment and making the accumulation of terpenoids either more or less likely depending on the level of light penetration (Fig. 7).

C_6_ volatiles are biosynthesized from linoleic and linolenic fatty acid precursors via lipoxygenases (Schwab *et al.*, 2008). The C_6_ aldehyde (E)-2-hexenal was the most abundant volatile that we detected in all berry tissues and genotypes, in agreement with reports in other grapevine cultivars (Kalua and Boss, 2010; Qian *et al.*, 2017; Maoz *et al.*, 2020), and it consistently showed greater accumulation in the white-berried variants of both Tempranillo and Garnacha (Supplementary Table S7), supporting it as being a feature of white grapes. Its increased accumulation in white grapes might be light-dependent given that C_6_ volatiles increase after defoliation of grapes (Piombino *et al.*, 2022), and also in the presence of high UV radiation (Joubert *et al.*, 2016). The lipoxygenase-encoding gene *VviLOXA* (Vitvi06g00158 = VIT_06s0004g01510) could participate in this response given that it has been defined as a ‘ripening switch’ gene specific to white-berried cultivars (Massonnet *et al.*, 2017), its expression correlates with the levels of C_6_ aldehydes in different cultivars (Qian *et al.*, 2017), and it was up-regulated in the skin of TB compared to TT from the veraison stage upon anthocyanin accumulation in the latter (Supplementary Fig. S8; Supplementary Table S4).

In contrast to C_6_ aldehydes, the most abundant C_6_ alcohols, 1-hexanol and (E)-2-hexen-1-ol, were detected at lower levels (>6.8-fold) in the white-berried variants in both the pericarp tissues, as was the ester (Z)-3-hexenyl acetate (>3.7-fold), which is derived from C_6_ alcohols (Supplementary Fig. S8; Supplementary Table S7). While alcohol dehydrogenases (ADHs) catalyse the reversible conversion of C_6_ aldehydes to the corresponding C_6_ alcohols (Schwab *et al.*, 2008), the down-regulation of an *ADH* gene (Vitvi14g02621 = VIT_14s0030g01030) that we detected in both the skin and flesh of TB compared to TT might have been related to the higher retention of aldehydes in the white-berried variants (Supplementary Fig. S8; Supplementary Table S4). Whilst higher C_6_ aldehyde to alcohol ratios have been observed when individual random pairs of white versus anthocyanin-pigmented cultivars have been compared (Kalua and Boss, 2010; Rambla *et al.*, 2016), our consistent results in two near-isogenic somatic variants and in flesh that lacked anthocyanins in black-berried variants, in addition to berry skin, provide for the first time strong support for the idea that alterations in the partitioning of fatty acid-derived volatiles are an indirect consequence of variation in berry colour. A higher temperature in anthocyanin-pigmented grapes during daytime throughout ripening (Fig. 2; Supplementary Fig. S2) might also be related to an increased metabolization of C_6_ compounds towards alcohols (Joslin and Ough, 1978). The higher ratio of aldehydes to alcohols could lead to a character of greater herbaceous aroma in white grapes (Wu *et al.*, 2016).

In line with the differences observed between ripening white and anthocyanin-pigmented grapes, variation in berry skin anthocyanin levels across black-skinned cultivars might also result in different berry light penetration and aroma potential, considering that the lower content of anthocyanins in the skin of GT compared to TT black-berried cultivars (Fig. 1F) correlated with the detection of higher levels of glycosylated and free monoterpenes as well as of the C_6_ aldehyde (E)-2-hexenal in GT than in TT grapes (Supplementary Fig. S9; Supplementary Table S7). Poorer anthocyanin sunscreening in GT compared with TT grapes might at least in part explain why the up-regulation of light-responsive genes was milder between GB and GT than between TB and TT, and probably also why the profile of volatile and aroma precursors of GT was intermediate between TT and the white-berried variants (Supplementary Fig. S6). Indeed, negative correlation of cultivar skin anthocyanin content with the expression of photosynthesis and radiation-responsive genes has also been described among red-berried cultivars (Massonnet *et al.*, 2017), further suggesting that these responses depend on anthocyanin levels changing the berry microenvironment (Fig. 7). Although our results support the idea that these differences in gene expression originate from variations in berry colour, and confirm their consequences in grape composition for the first time, there remains a need for further specific comparisons of light penetration and quantification of metabolites in grape-colour variants both before and after the onset of anthocyanin accumulation in the black-berried variants.

### Activation of phenylpropanoid biosynthesis and trihydroxylation in the presence of functional MYBA1 and MYBA2

Our results showed that an absence of functional *VviMYBA1* and *VviMYBA2* genes did not exclusively affect the accumulation of anthocyanins, but that other phenylpropanoid pathway branches were also down-regulated in the skin of the white-berried somatic variants (Fig. 4; Supplementary Fig. S3; Supplementary Table S4). This pattern affected genes that are also down-regulated in the pericarp of white compared to red grape cultivars, including *PAL*, *4CL*, and *C4H* at the base of the pathway,; as well *CHS*, *CHI*, *F3H*, *DFR*, *LDOX, AM3*, *3AT* (Vitvi03g01816 = VIT_03s0017g00870), and *GST4* that are involved in the biosynthesis of the flavonoid skeleton and the accumulation of anthocyanins into the vacuole (Castellarin *et al.*, 2006; Xie *et al.*, 2015; Massonnet *et al.*, 2017) (Fig. 4; Supplementary Table S4). Transgenic grapes or hairy roots overexpressing *VviMYBA1* also overexpress genes encoding PAL, CHS, F3H, LDOX, UFGT, the anthocyanin acyltransferase 3AT, and the anthocyanin vacuole transporters GST4 and AM3 (Cutanda-Perez *et al.*, 2009; Gomez *et al.*, 2011; Rinaldo *et al.*, 2015; Matus *et al.*, 2017), suggesting that they are all either direct targets of MYBA1 and/or MYBA2, as previously demonstrated for *Vvi3AT* and *VviUFGT* (Bogs *et al.*, 2007; Rinaldo *et al.*, 2015; Matus *et al.*, 2017), or indirect targets.

Our results also showed that functional MYBA1-MYBA2 are required for trihydroxylation not only of anthocyanins, as previously reported (Castellarin *et al.*, 2006; Xie *et al.*, 2015), but also of colourless flavonoids such as flavonols, as supported by the concurrent down-regulation of *F3´5´H* genes and the lack of accumulation of trihydroxylated flavonols in the skin of the white-berried variants (Fig. 4; Supplementary Figs S3, S4). Direct activation of an *F3´5´H* promoter by MYBA1 and MYBA2 has indeed been reported (Matus *et al.*, 2017), as well as the lack of *F3´5´H* expression in white-berried genotypes (Bogs *et al.*, 2006; Castellarin *et al.*, 2006; Rinaldo *et al.*, 2015; Massonnet *et al.*, 2017). This function of MYBA1-MYBA2 would explain the lack of trihydroxylated flavonol accumulation that has been described previously in different white-berried genotypes (Mattivi *et al.*, 2006; Ferreira *et al.*, 2016).

### Activation of aromatic and branched-chain amino acid consumption in the presence of functional MYBA1 and MYBA2

According to the consistent differences that we observed between the berry-colour somatic variants of both Tempranillo and Garnacha, a greater accumulation of the phenylpropanoid precursor aromatic amino acids phenylalanine and tyrosine as well as of BCAAs appears to be another feature of white grapes (Fig. 5; Supplementary Table S2). Comparable differences have previously been observed for phenylalanine between cultivars differing in grape colour (Degu *et al.*, 2015), and over-accumulation of aromatic amino acids and BCAAs have indeed been reported when the phenylpropanoid pathway is inhibited in transgenic poplar leaves (Ma *et al.*, 2018), suggesting that it is probably a consequence of decreased activity in the phenylpropanoid biosynthesis pathway.

Our RNA-seq results showed that the ‘Monocarboxylic acid metabolic process’ functional category was over-represented in genes from clusters TS 2 and GS 5, following a gene expression pattern of lower up-regulation during ripening in the white- than black-berried variants in both Tempranillo and Garnacha (Fig. 3C; Supplementary Tables S4, S5). This over-representation in both cultivars consisted of genes encoding two PALs, one pyruvate kinase (Vitvi08g01664 = VIT_08s0007g05490), one acetyl-CoA carboxylase 2 (*ACC2*, Vitvi18g00368 = VIT_18s0001g04980), and one biotin synthase, with lower expression in white-berried variants from the V stage when anthocyanin accumulation occurred in the black-berried variants (Fig. 3C; Supplementary Tables S4, S5), which was probably related to a lower consumption of aromatic amino acids, BCAAs, and derived acetyl-CoA donors in phenylpropanoid biosynthesis (Nikolau *et al.*, 2003). Supporting this possibility, one gene encoding a branched-chain alpha-keto acid dehydrogenase (Vitvi18g01174 = VIT_18s0001g14980) involved in the catabolism of BCAAs towards acetyl-CoA was also down-regulated in the skin of GB compared to GT (Supplementary Table S4). While it remains unclear whether these genes are directly or indirectly activated by the action of MYBA1-MYBA2, our results indicate for the first time that lack of consumption of BCAAs and aromatic amino acids for biosynthesis of anthocyanin precursors is a feature of white grapes.

In yeast fermentation, phenylalanine is a precursor of phenylacetaldehyde and 2-phenylethanol, whereas BCAAs are precursors of isobutanol and isobutyric acid (Fairbairn *et al.*, 2017; Dai *et al.*, 2021). Given that even minor changes in the amino acid concentration of musts have a direct impact on the formation of aromatic compounds (Fairbairn *et al.*, 2017), the metabolization of the higher contents of phenylalanine and BCAA grape precursors to these volatiles might contribute to higher honey, cheese, and rose notes in white-berry wines (Gambetta *et al.*, 2014).

### Enhanced abiotic and biotic stress protection in anthocyanin-pigmented grapes

It can be considered that the accumulation of anthocyanins and other polyphenols in the grape skin has been selected to protect the seeds from mutagenic radiation, and that it exerts antioxidant and antibiotic functions (Steyn *et al*., 2002; Blanco-Ulate *et al*., 2015). The greater accumulation of trihydroxylated flavonols by the action of MYBA1-MYBA2 in anthocyanin-pigmented grapes (Fig. 4; Supplementary Fig. S4) could correspond to a similar mechanism, given that trihydroxylated myricetin displays a greater antioxidant capacity than quercetin dihydroxyflavonol and kaempferol monohydroxyflavonol (Gordon and Roedig-Penman, 1998; Pekkarinen *et al*., 1999).

In addition to protection from abiotic stress factors, our transcriptomic results indicate that the ontogenic activation of pathogen defences that takes place in the grapevine fruit skin upon berry ripening (Lijavetzky *et al.*, 2012; Agudelo-Romero *et al.*, 2015) is enhanced in the presence of functional *VviMYBA1* and *VviMYBA2* (Fig. 3C; Supplementary Table S5). For instance, three *WRKY* genes (*WRKY08*, Vitvi04g00511 = VIT_04s0008g05760; *WRKY29*, Vitvi10g00063 = VIT_10s0116g01200; *WRKY47*, Vitvi15g01003 = VIT_15s0046g01140) that have been reported as being up-regulated in the grape skin during ripening (Lijavetzky *et al.*, 2012) also showed a more prominent up-regulation in the skin of the black-berried variants (Supplementary Table S4). The overexpression of *VviLOXO* (Vitvi09g00085 = VIT_09s0002g01080) encoding lipoxygenase in the skin of black- compared to white-berried variants (Supplementary Fig. S8; Supplementary Table S4) could contribute to enhanced pathogen defences, given that this gene is induced by wounding and *Botrytis cinerea* infection in grape berries, and it has been suggested to be involved in the biosynthesis of JA precursors (Podolyan *et al.*, 2010; Blanco-Ulate *et al.*, 2015). Consistent with *VviLOXO* expression, black-berried grapes accumulated higher levels of the C_6_ compounds 1-hexanol and (Z)-3-hexenyl acetate (Supplementary Fig. S8; Supplementary Table S7), and the same trend was followed by JA, with a significant colour effect in Garnacha (Table 1). This might be related to the greater susceptibility to powdery mildew described for GB than for GT (Cabello Sáenz de Santamaría *et al.*, 2019).

Treatment with JA and warm climates lead to increased accumulation of 1-hexanol in grape berries (Xu *et al.*, 2015; Gimenez-Banon *et al.*, 2022), and both JA and (Z)-3-hexenyl acetate volatiles can trigger resistance to herbivorous insects in plants (Farag *et al.*, 2005). Treatment with (Z)-3-hexenol, which can be rapidly metabolized to acetylated forms such as (Z)-3-hexenyl acetate, enhances resistance to insect attack by increasing the GABA content in Arabidopsis leaves (Farag *et al*., 2005; Jiao *et al.*, 2022). Accordingly, the higher GABA levels that we detected in black-berried variants (Fig. 5D; Supplementary Table S2), might be part of the cascade triggered by volatiles related to (Z)-3-hexenyl acetate in the skin of black berries. Greater accumulation of 1-hexanol and GABA has indeed been reported in grapes and wines of TT compared to TB (Garde-Cerdán *et al.*, 2021a, 2021b). Higher berry temperature in black-berried variants (Fig. 2; Supplementary Fig. S2) could contribute to this cascade, given that GABA accumulation is increased by shading and by heat in grapevine berries (Pereira *et al.*, 2006; Sweetman *et al.*, 2014; Guan *et al.*, 2017). Although further functional studies are required to confirm the roles of *LOXO* and *LOXA* and of the fatty acid-derived compounds and GABA in grape berries, the transcriptional activation of stress-protection and JA-signalling genes also correlates with anthocyanin content in coloured grape cultivars (Massonnet *et al.*, 2017). This points to direct or indirect activation of pathogen defences by the action of MYBA1-MYBA2 in anthocyanin-pigmented grapes.

### Conclusions

Our results indicate that in addition to determining the dark colour of berries by triggering anthocyanin accumulation, the transcription factors MYBA1 and MYBA2 modulate other important grape features. These are related to the direct action of MYBA1-MYBA2 on colourless compounds, such as on the partitioning of flavonol hydroxylation; metabolic side-effects that include increased consumption of phenylpropanoid precursor amino acids as acetyl-CoA donors; and indirect effects on the internal microenvironment of the berry due to the absorbance of light by anthocyanins and consequent heating and shading, which affects the accumulation of aroma precursors such as terpenes and volatiles derived from fatty acids, and signalling molecules such as GABA and JA (Fig. 7). Our findings show that these effects could determine the differences in flavours and stress responses that characterize white and anthocyanin-pigmented grapes, although genomic regions specifically controlling these pathways will also contribute to the diversity in these features irrespective of grape colour (Lin *et al.*, 2019). Nevertheless, our results suggest that genetic variation at the grape-colour locus could be responsible for the QTLs for berry terpene content that have repeatedly been reported on LG2 (Doligez *et al.*, 2006b; Battilana *et al.*, 2009; Duchene *et al.*, 2009; Koyama *et al.*, 2022), and this has not been considered before. In addition to direct effects in the berry skin, our findings indicate indirect consequences of variation in grape skin colour altering the composition of the berry flesh, and these effects could have an impact on wine composition, even in white wine production in which extraction of skin compounds is limited. The effects on the grape microenvironment that we have found to be associated with the presence of anthocyanins could be generally expected in other species as well, and hence our findings suggest that similar side-effects on fruit composition to the ones reported here might occur in other edible fleshy fruit crops that show fruit colour diversity due to variation in anthocyanin pigmentation.

## Supplementary data

The following supplementary data are available at *JXB* online.

Table S1. Details of berry sampling.

Table S2. Contents of the sugar, organic acid, and amino acid primary metabolites examined the berry skin and flesh tissues of the grape-colour somatic variants.

Table S3. Berry temperatures in the TB and TT somatic variants.

Table S4. Differentially expressed genes between the berry-colour somatic variants identified by RNA-seq.

Table S5. Functional enrichment in the clusters of differentially expressed genes.

Table S6. Phytohormone contents in the berry skin of the grape-colour somatic variants.

Table S7. Aroma precursor and volatile metabolite contents in berry skin and flesh tissues of the grape-colour somatic variants.

Fig. S1. Cluster number estimation of RNA-seq differentially expressed genes by hierarchical clustering.

Fig. S2. Mean hourly berry temperature estimated over the post-veraison, mid-ripening, and pre-harvest periods.

Fig. S3. Gene expression heatmap of differentially expressed genes in the phenylpropanoid biosynthesis pathway.

Fig. S4. Berry skin flavonol contents in the grape-colour somatic variants.

Fig. S5. Gene expression heatmap of differentially expressed genes located in genome regions that are hemizygous in Tempranillo Blanco due to somatic genome rearrangement.

Fig. S6. PCA and berry-colour factor PLS-DA plots based on berry aroma precursor and volatile metabolite composition in the black- and white-berried somatic variants.

Fig. S7. PCA and berry-colour factor PLS-DA plots based on amino acid composition in the black- and white-berried somatic variants.

Fig. S8. Diagram of transcriptomic and berry composition effects of grape colour variation on fatty acid-derived C_6_ compounds.

Fig. S9. PLS-DA VIP scores for cultivar factor based on berry aroma precursor and volatile metabolite composition in the black-berried Tempranillo and Garnacha cultivars.

erad223_suppl_Supplementary_Table_S1_Figures_S1-S9

erad223_suppl_Supplementary_Table_S2

erad223_suppl_Supplementary_Table_S3

erad223_suppl_Supplementary_Table_S4

erad223_suppl_Supplementary_Table_S5

erad223_suppl_Supplementary_Table_S6

erad223_suppl_Supplementary_Table_S7

## Data Availability

The raw RNA-seq dataset for this study has been deposited in the European Nucleotide Archive (ENA) at EMBL-EBI. https://www.ebi.ac.uk/ena/browser/view/PRJEB60837.

## Abbreviations:

BCAAs: branched-chain amino acids;
DEG: differentially expressed gene;
FPKM: fragments per kb per million counts;
GABA: γ-aminobutyric acid;
GB: Garnacha Blanca;
GF: Garnacha berry flesh developmental series;
GS: Garnacha berry skin developmental series;
GT: Garnacha Tinta;
JA: jasmonic acid;
LG2: linkage group 2;
M: maturity or full ripening;
PV: pre-veraison;
PM: pre-mature ripening;
QTL: quantitative trait locus;
TB: Tempranillo Blanco;
TF: Tempranillo berry flesh developmental series;
TS: Tempranillo berry skin developmental series;
TT: Tempranillo Tinto;
PCA: principal component analysis;
PLS-DA: partial least squares-discriminant analysis;
SA: salicylic acid;
STS: stilbene synthase;
UFGT: UDP-glucose:flavonoid 3-O-glucosyltransferase;
V: veraison;
VIP: variable importance in projection.
