# Supplementary material for: The flavour of grape colour: anthocyanin content tunes aroma precursor composition by altering the berry microenvironment
Source: J Exp Bot. 2023 Jun 9;74(20):6369–90. doi: 10.1093/jxb/erad223 (PMC10627162; doi:10.1093/jxb/erad223)
Supplement: erad223_suppl_Supplementary_Table_S1_Figures_S1-S9 [file erad223_suppl_supplementary_table_s1_figures_s1-s9.pdf]

Supplementary Files

The flavour of grape color: anthocyanin content tunes aroma precursor composition by altering the berry microenvironment

Authors

Maite Rodríguez-Lorenzo[1], Nuria Mauri[1], Carolina Royo[1], José L. Rambla[2,3], Gianfranco Diretto[4], Olivia Demurtas[4], Ghislaine Hilbert[5], Christel Renaud[5], Vanessa Tobar[6], Joaquín Huete[6], Serge Delrot[5], Antonio Granell[2], José Miguel Martínez-Zapater[1], Pablo Carbonell-Bejerano[1]

[1]Instituto de Ciencias de la Vid y del Vino, ICVV, CSIC - Universidad de La Rioja - Gobierno de La Rioja, 26007 Logroño, Spain

[2]Instituto de Biología Molecular y Celular de Plantas, IBMCP, CSIC - Universidad Politécnica de Valencia, 46011 Valencia, Spain.

[3]Universitat Jaume I, Departamento de Biología, Bioquímica y Ciencias Naturales, 12071 Castellón de la Plana, Spain

[4]Italian National Agency for New Technologies Energy and Sustainable Development, Casaccia Research Centre, 00123 Rome, Italy

[5]EGFV, Bordeaux Sciences Agro, INRA - Université de Bordeaux, ISVV, 33140 Villenave d'Ornon, France

Servicio de Información Agroclimática de La Rioja (SIAR). Consejería de Agricultura, Ganadería y Medio Ambiente, Gobierno de La Rioja, 26007 Logroño, Spain

Supplementary Tables

Supplementary Table S1. Details of berry sampling. Correspondence of berry samples collected at pre-veraison (PV), veraison (V), pre-maturity (PM) and maturity (M) developmental stages in 2016 and 2017 seasons for transcriptomics and metabolomics analyses in Tempranillo Tinto (TT), Tempranillo Blanco (TB), Garnacha Tinta (GT) and Garnacha Blanca (GB) berry colour somatic variants. The sampling date, ripening state, selected berry density interval by floatation in NaCl solutions, the berry juice total soluble solids (TSS) estimated with by refractometer and the use given to berries from each sampling date is indicated.

Stage ID	Genotype	Sampling date	Selected berries	Density [g NaCl·L-1]	TSS [ºBrix]	RNA-seq	Precursor and volatiles	Hormone	Primary compounds and flavonols	Anthocyanins in TT and GT
PV16	TT, TB	04/08/2016	hard green	-	-	04/08/2016				
	GT, GB	12/08/2016	hard green	-	-	12/08/2016				
PV17	TT, TB	21/07/2017	hard green	-	-				21/07/2017	
	GT, GB	27/07/2017	hard green	-	-				27/07/2017	
V16	TT, TB, GT, GB	18/08/2016	ripening	90 - 100	14.4 ± 0.9	18/08/2016				
V17	TT, TB, GT, GB	03/08/2017	ripening	110 - 120	16.4 ± 0.5				03/08/2017	03/08/2017
PM16	TT, TB, GT, GB	06/09/2016	ripening	120 - 130	20.6 ± 0.4	06/09/2016		06/09/2016		
PM17	TT, TB, GT, GB	17/08/2017	ripening	120 - 130	20.6 ± 0.4				17/08/2017	17/08/2017
M16	TT, TB, GT, GB	13/09/2016	ripening	140 - 160	23.0 ± 0.3		13/09/2016		13/09/2016	13/09/2016
M17	TT, TB, GT, GB	31/08/2017	ripening	140 - 160	23.3 ± 0.2				31/08/2017	31/08/2017

Supplementary Figures

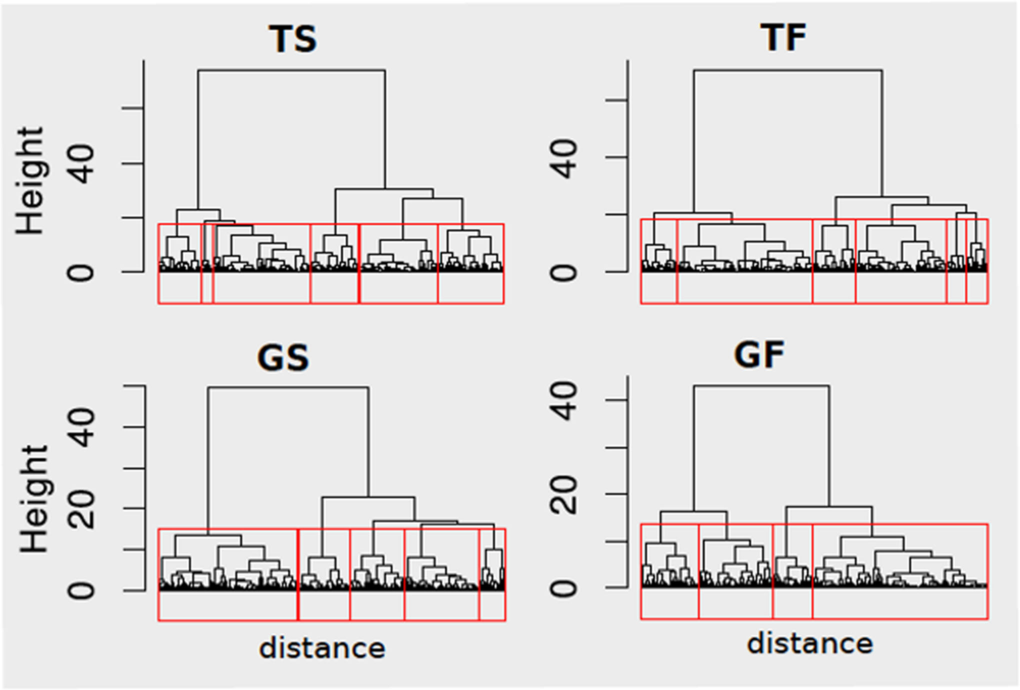

Supplementary Figure S1. Cluster number estimation of RNA-seq differentially expressed genes by hierarchical clustering. To estimate the optimal number of clusters of differentially expressed genes (DEGs) on each of the RNA-seq ripening series of white- versus black-berried somatic variants, a hierarchical clustering analysis using the agglomeration method "ward.D2" implemented in *hclust* (stats) function was conducted. The ripening series comprised pre-verasion, veraison and pre-maturity developmental stages for Tempranillo berry skin (TS), Tempranillo berry flesh (TF), Garnacha berry skin (GS), and Garnacha berry flesh (GF). DEGs were identified according to <0.05% FDR in masigpro two-class time series and ≥1.75-fold change in at least one stage. Red rectangles indicate the number of clusters identified on the dendrogram for each series.

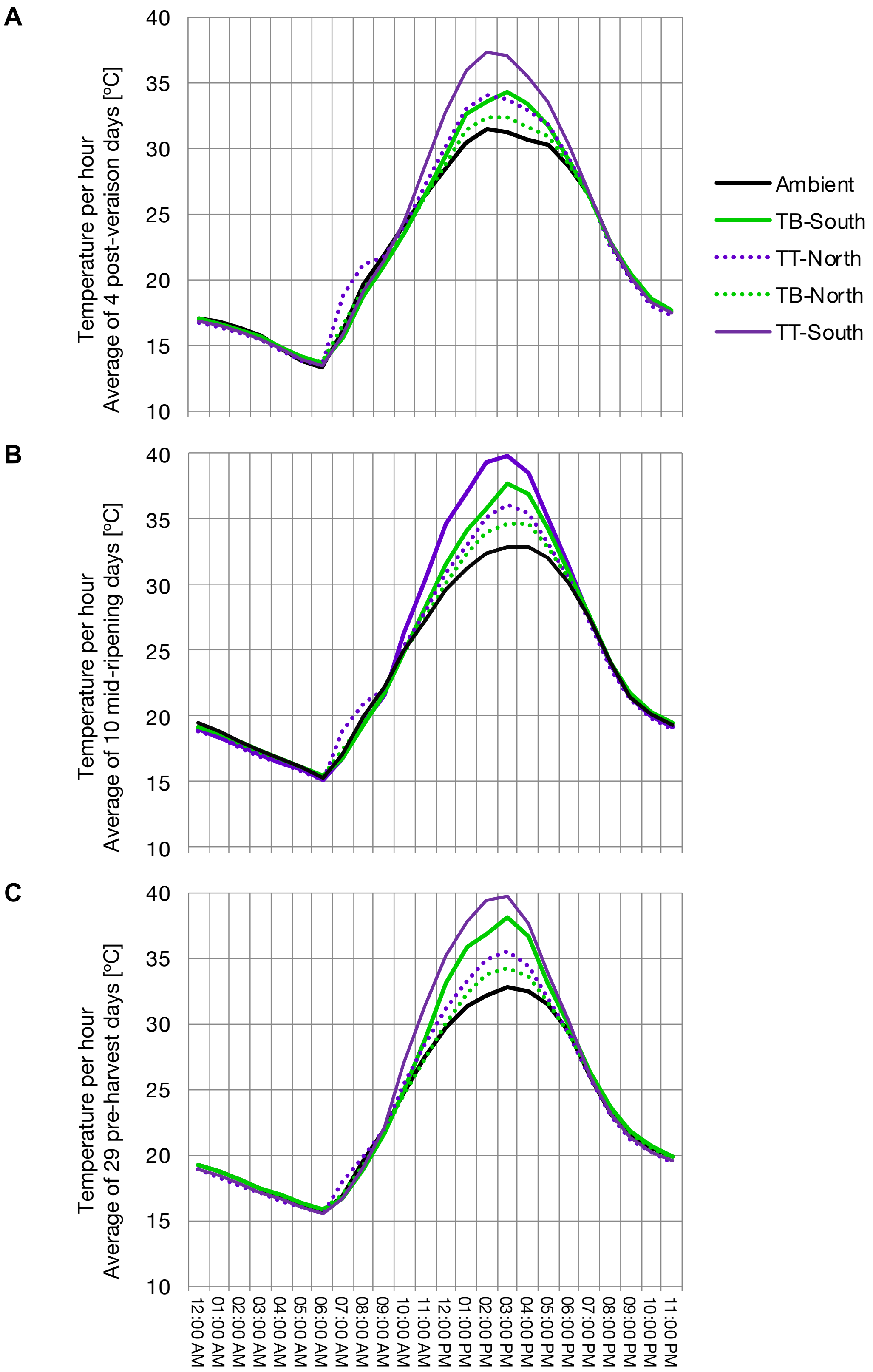

Supplementary Figure S2. Mean berry temperature per hour estimated over post-veraison, mid-ripening and pre-harvest periods. Average temperature per hour is depicted for Tempranillo Blanco (TB) and Tempranillo Tinto (TT) berries along with the ambient air temperature in the periods of a) four post-veraison (2016/08/11 - 2016/08/15), b) ten mid-ripening (2016/08/16 - 2016/08/26), and c) 29 pre-harvest days (2016/08/17 - 2016/09/15).

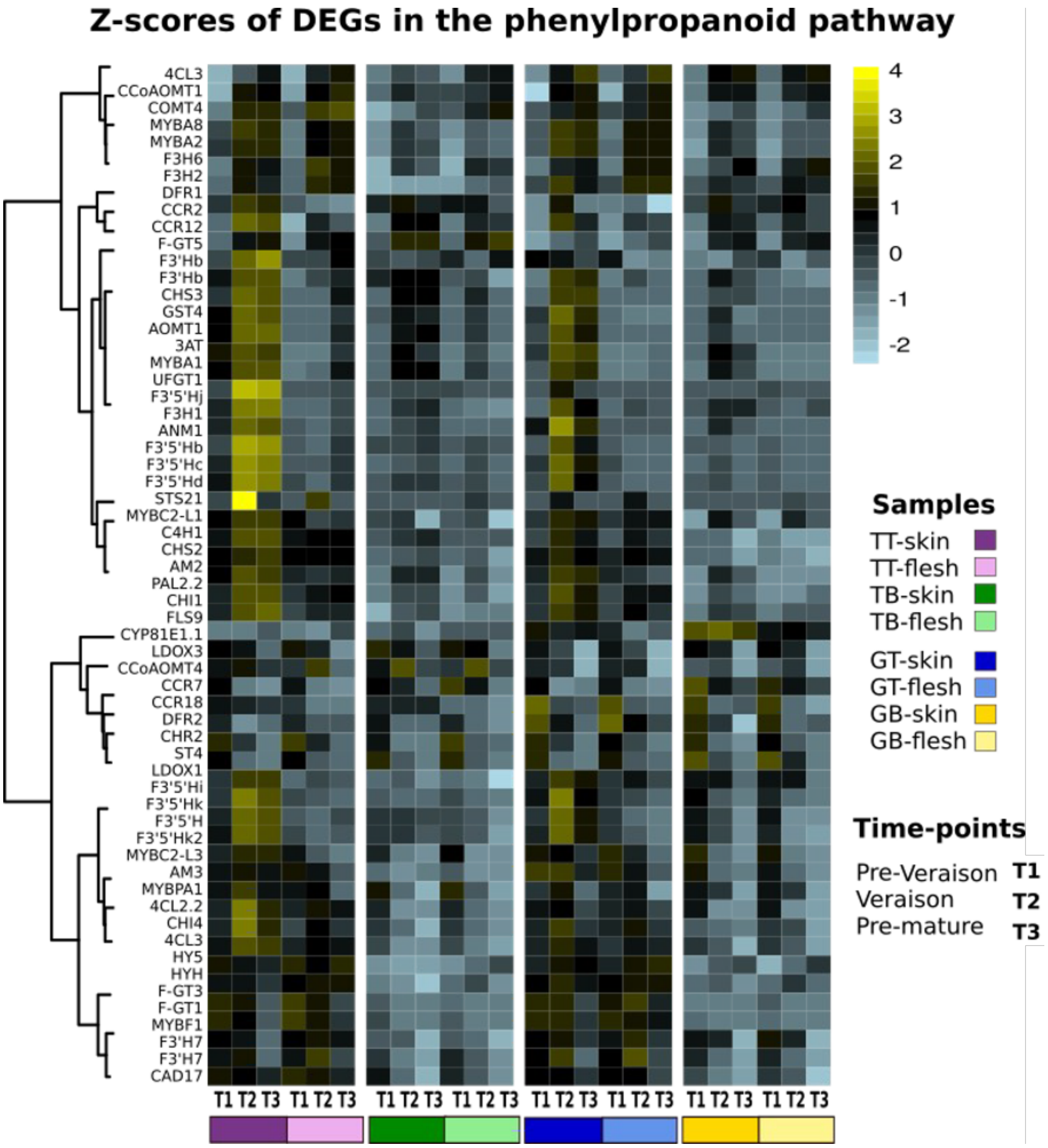

Supplementary Figure S3. Gene expression heatmap of differentially expressed genes in the phenylpropanoid biosynthesis pathway. Heatmap for normalized *z*-score FPKM of RNA-seq counts in all four berry ripening series is displayed for genes with phenylpropanoid pathway-related functional annotations. Genes are displayed if they were detected as differentially expressed gene (DEG) in at least one of the four series according to <0.05% FDR in masigpro two-class time series and ≥1.75-fold change in at least one stage. Pre-verasion, veraison and pre-maturity developmental z-score values averaged for three replicates per sample are depicted for the ripening series in Tempranillo Tinto berry skin (TT-skin), Tempranillo Tinto berry flesh (TT-flesh), Tempranillo Blanco berry skin (TB-skin), Tempranillo Blanco berry flesh (TB-flesh), Garnacha Tinta berry skin (GT-skin), Garnacha Tinta berry flesh (GT-flesh), Garnacha Blanca berry skin (GB-skin), and Garnacha Blanca berry flesh (GB-flesh). Genes are ordered according to a hierarchical clustering of expression values obtained using the agglomeration method "ward.D2" implemented in *hclust*.

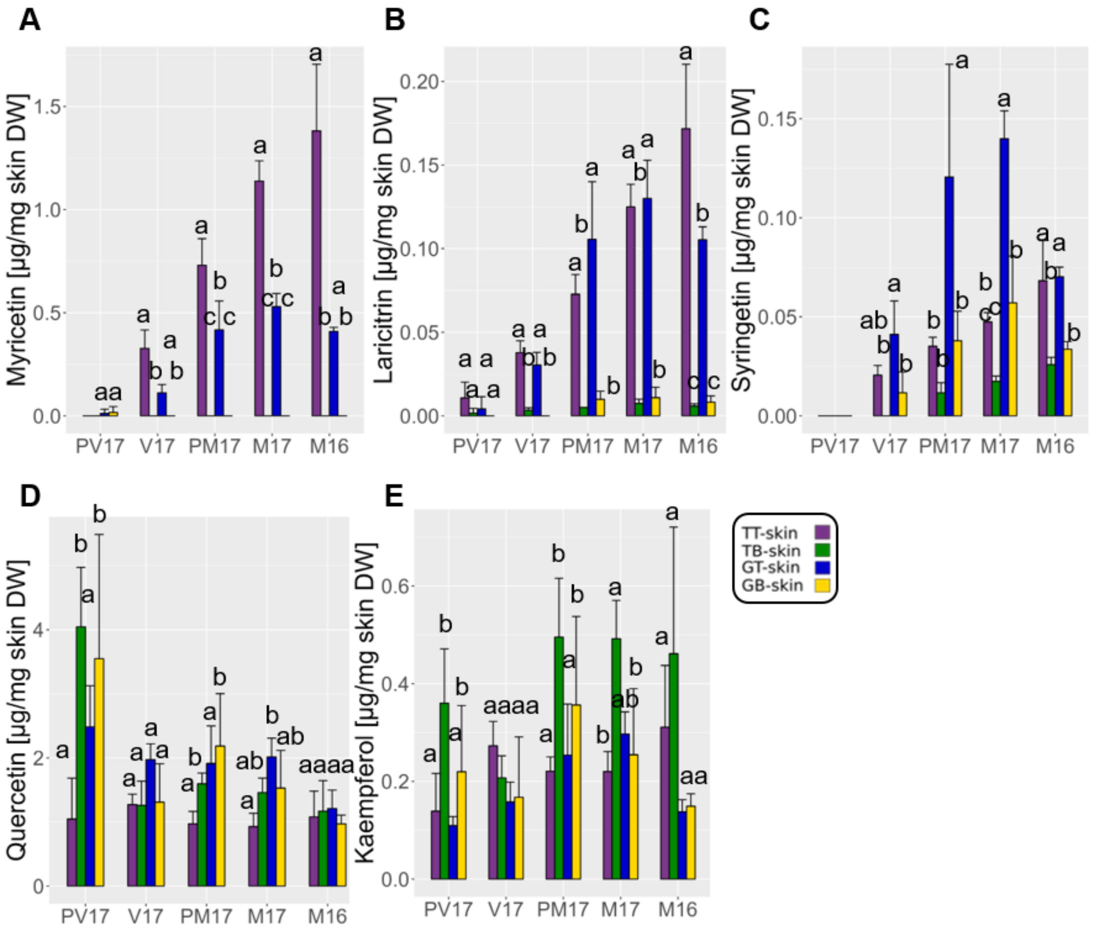

Supplementary Figure S4. Berry skin flavonol contents in the grape-colour somatic variants. Content of a) myricetin, b) laricitrin, c) syringetin, d) quercetin, and e) kaempferol in berry skin of Tempranillo Tinto (TT), Tempranillo Blanco (TB), Garnacha Tinta (GT) and Garnacha Blanca (GB) for pre-veraison (PV17), veraison (V17) and maturity (M17) developmental stages from 2017 season along with maturity stage from 2016 season (M16). At each stage, significant differences for metabolite content were considered according to *P* <0.05 in ANOVA and 'abc' characters denote differential groups according to HSD-Tukey posthoc test (*P* <0.05).

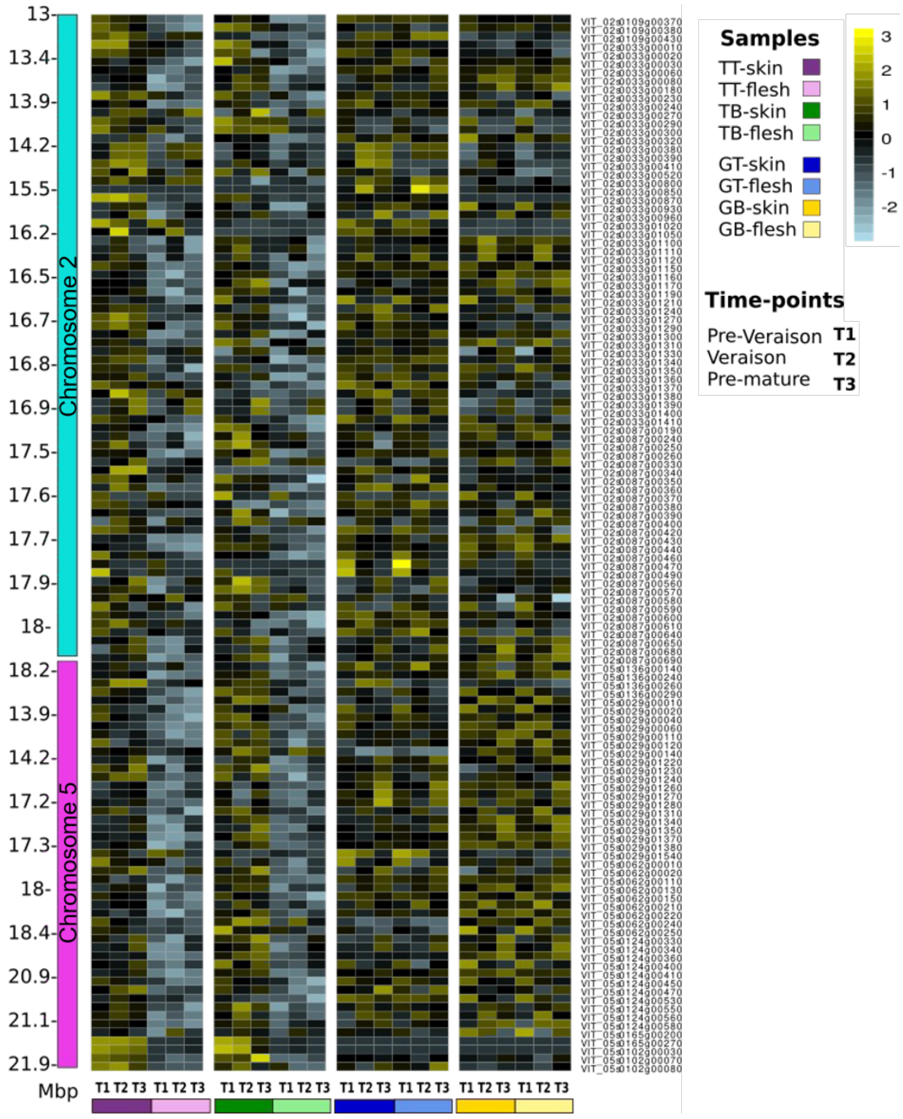

Supplementary Figure S5. Gene expression heatmap of differentially expressed genes located in genome regions that are hemizygous in Tempranillo Blanco due to somatic genome rearrangement. Heatmap for normalized *z*-score FPKM of RNA-seq counts in all four berry ripening series is displayed for genes with phenylpropanoid pathway-related functional annotations. Genes are displayed if they collocate with chromosome 2 and chromosome 5 regions identified as hemizygous in Tempranillo Blanco (Carbonell-Bejerano et al., Plant Physiology 2017, 175: 786-801) and were detected as differentially expressed gene (DEG) in the current study. DEGs were considered here according to <0.05% FDR in masigpro two-class time series and ≥1.75-fold change in at least one stage and in one out of four ripening series. Pre-verasion, veraison and pre-maturity developmental z-score values averaged for three replicates per sample are depicted for the ripening series in Tempranillo Tinto berry skin (TT-skin), Tempranillo Tinto berry flesh (TT-flesh), Tempranillo Blanco berry skin (TB-skin), Tempranillo Blanco berry flesh (TB-flesh), Garnacha Tinta berry skin (GT-skin), Garnacha Tinta berry flesh (GT-flesh), Garnacha Blanca berry skin (GB-skin), and Garnacha Blanca berry flesh (GB-flesh). Genes are ordered according to a hierarchical clustering of expression values obtained using the agglomeration method "ward.D2" implemented in *hclust*.

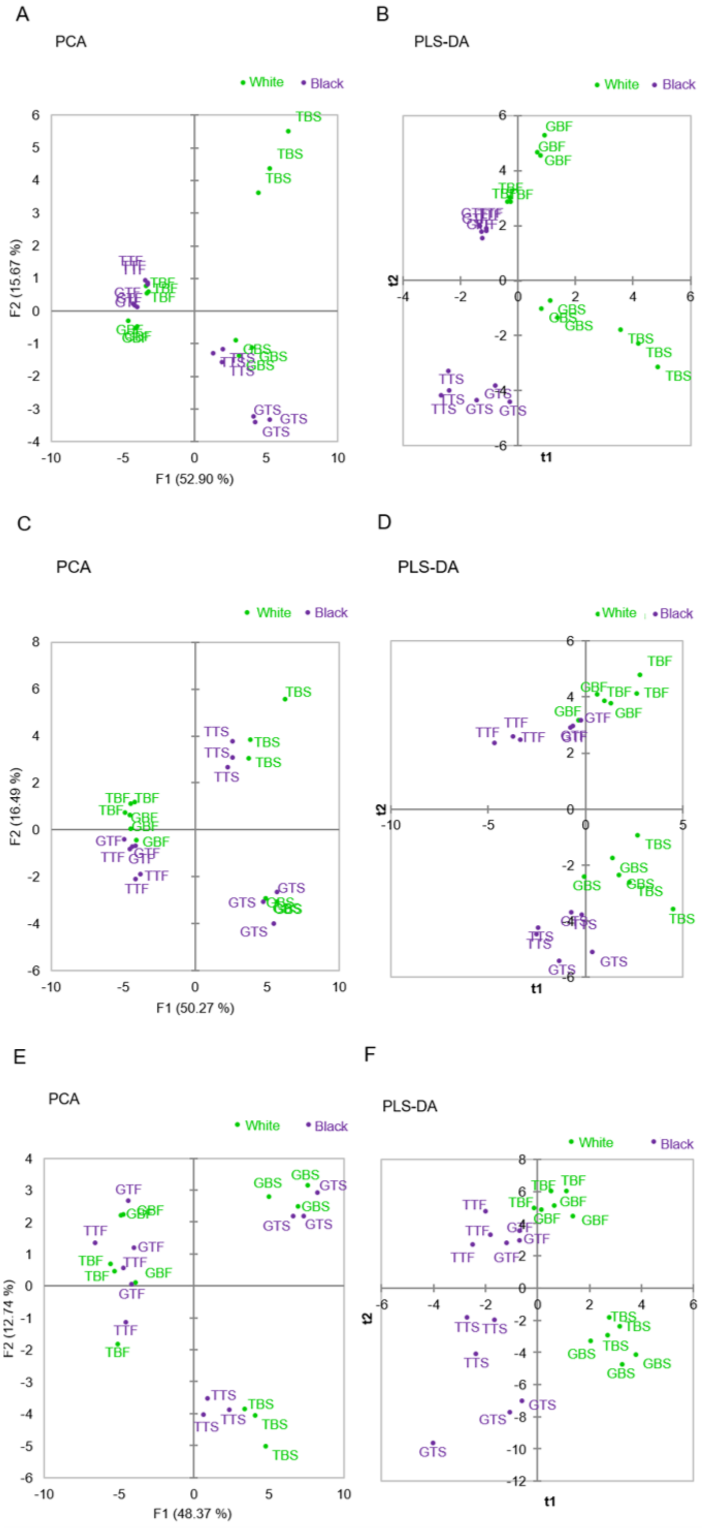

Supplementary Figure S6. PCA and berry colour factor PLS-DA plots based on berry aroma precursor and volatile metabolite composition in the black- and white-berried somatic variants. Principal component analysis (PCA) and berry colour factor partial least square discriminant analysis (PLS-DA) results, respectively, are shown for non-polar compounds in a) and b), semi-polar aroma precursors in c) and d), and volatile compounds in e) and f). For each type of compound, berry skin and flesh obtained from samples collected at maturity in 2016 season were analysed together. Samples are depicted in colour according to berry white (green) or coloured (purple) somatic variants.

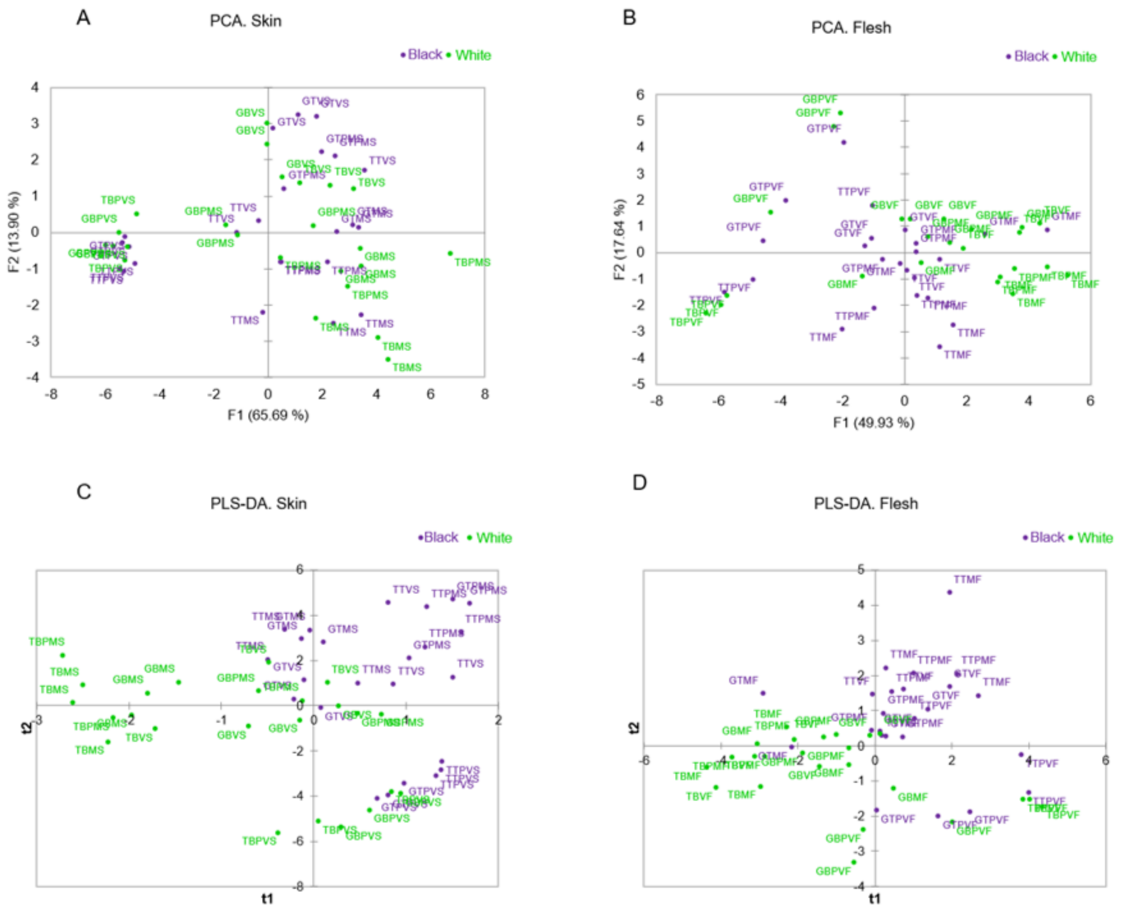

Supplementary Figure S7. PCA and berry-colour factor PLS-DA plots based on amino acid composition in the black- and white-berried somatic variants. Principal component analysis (PCA) and berry colour factor partial least square discriminant analysis (PLS-DA) results from Tempranillo Tinto (TT), Tempranillo Blanco (TB), Garnacha Tinta (GT) and Garnacha Blanca (GB) samples from pre-veraison (PV), veraison (V) and maturity (M) developmental stages collected during 2017 season are shown. a) PCA for berry skin, b) PCA for berry flesh, c) PLS-DA for berry skin, b) PLS-DA for berry flesh. Samples are depicted in colour according to berry white (green) or coloured (purple) somatic variants.

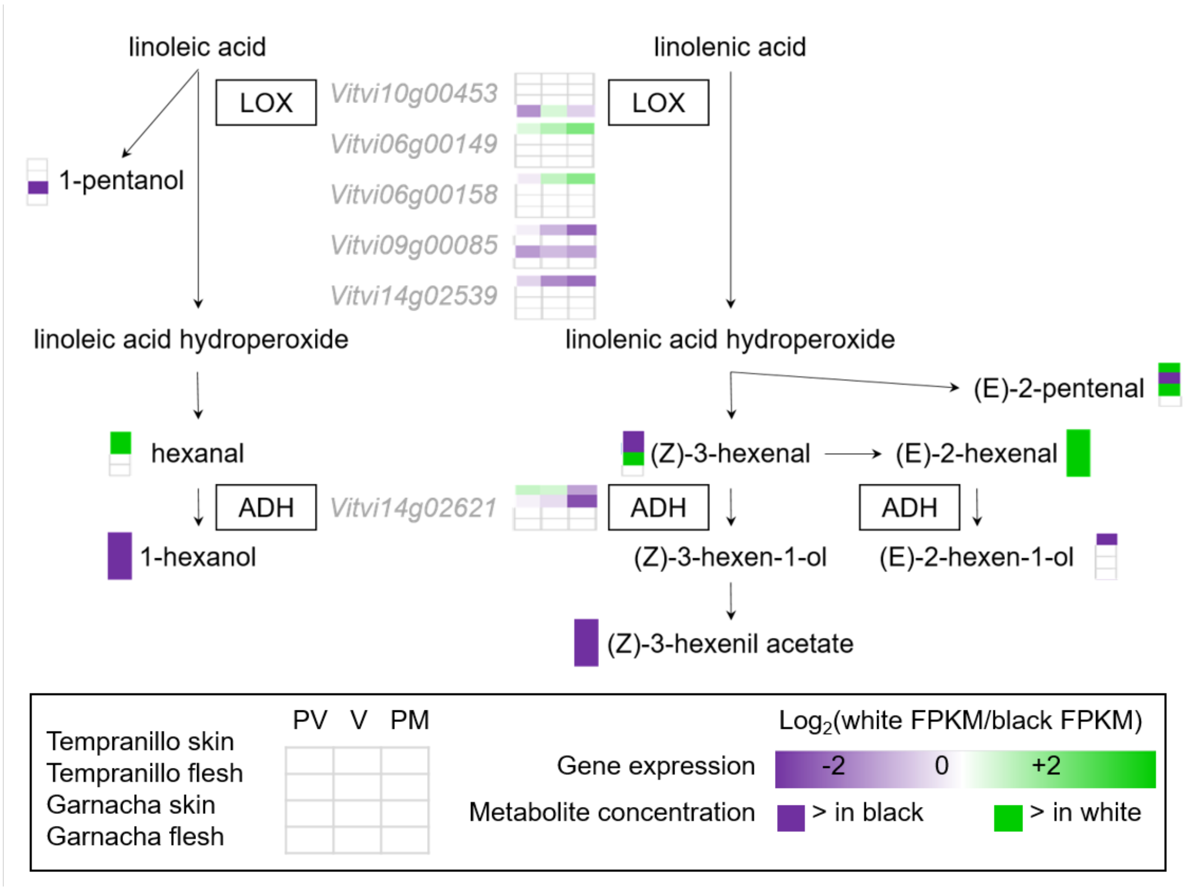

Supplementary Figure S8. Diagram of transcriptomic and berry composition effects of grape colour variation on fatty acid-derived C6 compounds. A diagram of C6 compounds pathway is shown. The abbreviated name of DEGs (differentially expressed genes) identified in the RNA-seq are boxed. Heatmaps representing DEG expression ratio in white-to-black berry somatic variants in Tempranillo and Garnacha and in skin and flesh developmental series (PV, pre-veraison; V, veraison; PM, pre-maturity) are depicted. DEGs were considered according to <0.05% FDR in masigpro two-class time series and ≥1.75-fold change in at least one stage. LOX: lipoxygenase, ADH: aldehyde dehydrogenase. In case of significant differences for metabolite levels at the analysed stage (maturity of 2016 season), the direction of the difference is indicated in a heatmap. Significant differences for metabolite content were considered according to *P* <0.05 in t-test or VIP >1 for colour factor PLS-DA.

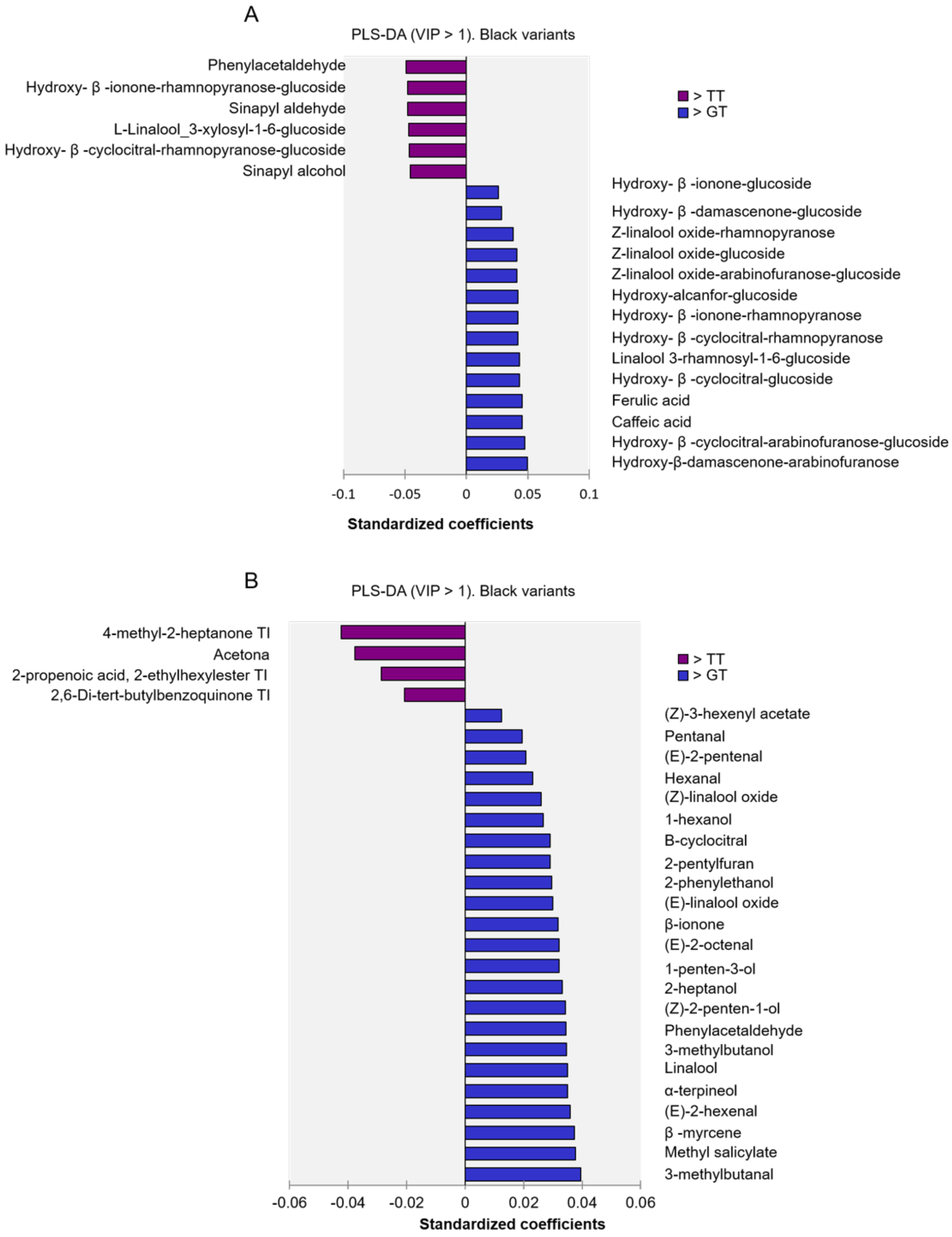

Supplementary Figure S9. PLS-DA VIP scores for cultivar factor based on berry skin aroma precursor and volatile metabolite composition in black-berried Tempranillo and Garnacha cultivars. Representation of the standardized coefficient of the compounds with VIP >1 in the PLS-DA analysis for cultivar genotype factor carried out for a) semi-polar precursors and b) volatiles in Tempranillo Tinto (TT) and Garnacha Tinta (GT) black-berried cultivars considering berry skin samples collected at maturity in 2016 season. Positive and negative PLS-DA coefficients indicate, respectively, for higher and lower metabolite accumulation in GT and TT, respectively.
